# Mitochondrial transfer from bone mesenchymal stem cells protects against tendinopathy both in vitro and in vivo

**DOI:** 10.1186/s13287-023-03329-0

**Published:** 2023-04-26

**Authors:** Bing Wei, Mingliang Ji, Yucheng Lin, Shanzheng Wang, Yuxi Liu, Rui Geng, Xinyue Hu, Li Xu, Zhuang Li, Weituo Zhang, Jun Lu

**Affiliations:** 1 School of Medicine, Southeast University, No. 87 Dingjiaqiao Road, Gulou District, Jiangsu Province 210009 Nanjing, People’s Republic of China; 2 Department of Orthopaedic Surgery/Joint and Sports Medicine Center, Zhongda Hospital, Southeast University, Nanjing, 210009 Jiangsu Province People’s Republic of China

**Keywords:** Mesenchymal stem cells, Achilles tendinopathy, Mitochondrial transfer, Tenocytes, Mitochondrial dysfunction, Apoptosis

## Abstract

**Background:**

Although mesenchymal stem cells (MSCs) have been effective in tendinopathy, the mechanisms by which MSCs promote tendon healing have not been fully elucidated. In this study, we tested the hypothesis that MSCs transfer mitochondria to injured tenocytes in vitro and in vivo to protect against Achilles tendinopathy (AT).

**Methods:**

Bone marrow MSCs and H_2_O_2_-injured tenocytes were co-cultured, and mitochondrial transfer was visualized by MitoTracker dye staining. Mitochondrial function, including mitochondrial membrane potential, oxygen consumption rate, and adenosine triphosphate content, was quantified in sorted tenocytes. Tenocyte proliferation, apoptosis, oxidative stress, and inflammation were analyzed. Furthermore, a collagenase type I-induced rat AT model was used to detect mitochondrial transfer in tissues and evaluate Achilles tendon healing.

**Results:**

MSCs successfully donated healthy mitochondria to in vitro and in vivo damaged tenocytes. Interestingly, mitochondrial transfer was almost completely blocked by co-treatment with cytochalasin B. Transfer of MSC-derived mitochondria decreased apoptosis, promoted proliferation, and restored mitochondrial function in H_2_O_2_-induced tenocytes. A decrease in reactive oxygen species and pro-inflammatory cytokine levels (interleukin-6 and -1β) was observed. In vivo, mitochondrial transfer from MSCs improved the expression of tendon-specific markers (scleraxis, tenascin C, and tenomodulin) and decreased the infiltration of inflammatory cells into the tendon. In addition, the fibers of the tendon tissue were neatly arranged and the structure of the tendon was remodeled. Inhibition of mitochondrial transfer by cytochalasin B abrogated the therapeutic efficacy of MSCs in tenocytes and tendon tissues.

**Conclusions:**

MSCs rescued distressed tenocytes from apoptosis by transferring mitochondria. This provides evidence that mitochondrial transfer is one mechanism by which MSCs exert their therapeutic effects on damaged tenocytes.

**Supplementary Information:**

The online version contains supplementary material available at 10.1186/s13287-023-03329-0.

## Background

Tendinopathy, previously known as tendinitis or tendinosis, is a common chronic musculoskeletal disorder clinically characterized by pain, swelling, and dysfunction, leading to tendon tear and rupture [1, 2]. Tendinopathy affects athletes and populations whose tendons are subjected to excessive or repetitive stretching [1]. Achilles tendinopathy (AT) is a highly prevalent condition, with an overall incidence rate of 2.35 per 1000 registered adults patients (mean age: 43.4 yeas) [3]. The underlying histopathology of tendinopathy involves an increased tenocyte response and a localized disorganization of the tendon structure [4]. Fortunately, stem cells-based therapy for various diseases has led to breakthroughs in the treatment of tendinopathy [5–9]. However, the exact mechanisms underlying the effects of stem cells on this condition remain unclear.

Mitochondria are important gatekeepers in the life and death of eukaryotic cells [10]. Mitochondrial dysfunction is associated with health problems, such as cancer, aging, metabolic, and cardiovascular disease. An increasing number of studies are being conducted that focus on mitochondria. Transferring exogenous mitochondria to cells is currently envisioned as a mechanism for stem cell-based therapy. Mesenchymal stem cells (MSCs) improve inflammation and cell death in several ways, including directed differentiation, exosomes, and paracrine activity [11–13]. A recently reported mechanism involves the donation of mitochondria from MSCs to recipient cells via intercellular transport mechanisms. Islam et al. [14] initially described a method for mediating the mitochondrial transfer from MSCs to damaged lung epithelial cells in 2012. Subsequently, the mitochondria of bone marrow mesenchymal stem cells (BMSCs) have been demonstrated to be transferred to cardiomyocytes [15], lung microvasculature endothelial cells [16], astrocytes [17], and airway smooth muscle cells [18]. Recipient cells of mitochondria from stem cells can resist oxidative stress, increase adenosine triphosphate (ATP) content and mitochondrial membrane potential (ΔΨm), promote proliferation, and reduce apoptosis [19, 20].

Tendinopathy is also associated with mitochondrial dysfunction [21, 22]. A study has shown that exogenous mitochondria were successfully transplanted into injured tenocytes and tendons and demonstrated a modulation of anti-inflammatory and apoptotic processes [23]. The therapeutic effect of MSCs applied to AT has been reported [8] and that in equine tendinopathy [9]. Goldberg et al*.* [24] conducted a single-center phase IIA proof-of-concept study of 10 patients who received BMSCs with AT repair. Six months after the procedure, all patients had improved clinical scores and the primary outcome was safety. This study aimed to test the hypothesis that BMSCs transfer mitochondria to tenocytes injured by H_2_O_2_ and collagenase-induced Achilles tendons, improving tenocyte function and health in vitro and in vivo.

## Methods

### Ethics statement

Male Sprague–Dawley rats (4–8 weeks old) were purchased from Beijing Weitong Lihua Experimental Animal Technology Co., Ltd. All experimental methods were conducted following the guidelines of the Committee of Animal Protection and Utilization of Southeast University and were handled according to protocols approved (approval no. 20210615006) by the Animal Research Ethics Committee of Southeast University (Nanjing, China).

### Primary cell extracts and cell culture

Primary bone marrow-derived MSCs were isolated from 4- to 6-week-old Sprague–Dawley rats using the whole bone marrow adherent method, as previously described [25]. Non-adherent cells were removed after 24 h, and MSCs were cultured to expand. The MSCs were cultured in Dulbecco’s modified Eagle's medium/F12 (DMEM/F12; Gibco, USA) supplemented with 10% fetal bovine serum (FBS; Gibco, Australia) and 1% penicillin/streptomycin (P/S; Gibco, USA) and incubated at 37 °C and 5% CO_2_. MSCs surface antigens at passage 2 were analyzed by flow cytometry (BD FACSCelesta). The MSCs were incubated with FITC-conjugated CD34 antibody (eBioscience), FITC-conjugated CD90 antibody (BD Biosciences), PE-conjugated CD45 antibody (BD Biosciences), and PE-conjugated CD44 antibody (eBioscience). The MSCs expressed CD90 and CD44 and were negative for CD45 and CD34 (Additional file 1: Fig S1A), as recently reported [26]. Furthermore, the trilineage differentiation potential of MSCs at passage 2 was assessed according to the manufacturer’s recommendations. Osteogenic, chondrogenic, and adipogenic differentiation were successfully induced in rat bone marrow MSCs complete induction medium (Cyagen, Suzhou, China) and photographed under an Olympus BX53 light microscope equipped with an Olympus SC100 digital camera (Olympus, Tokyo, Japan), and processed with the cellSens 2.0 Software (Olympus) (Additional file 1: Fig. S1B). Cells of passages 2–4 were used for follow-up experiments.

Primary tenocytes were isolated from 6- to 8-week-old Sprague–Dawley rats according to a previously described protocol [27]. Briefly, collected Achilles tendon tissues were cut into < 1 mm pieces and treated with 0.25% trypsin (Sigma-Aldrich), followed by digestion with 5 mg/mL type I collagenase (Solarbio, Beijing, China) in DMEM/F12 with 10% FBS at 37 °C for up to 6 h. Dissociated cells by a 70-μm-pore-size filter were used for centrifugal elutriation. Tenocytes were re-suspended in DMEM/F12 containing 10% FBS and 1% P/S. To confirm the characterization of the tenocytes, the expression of the matrix protein was examined by immunofluorescence (IF) staining with anti-collagen I or anti-collagen III antibodies (Servicebio, Wuhan, China). Consistent with a previous study [28], tenocytes expressed collagen type I (Col I) to an extent greater than collagen type III (Col III) (Additional file 2: Fig. S2A). Furthermore, as recently reported [28, 29], tenocytes were confirmed by reverse transcription and quantitative polymerase chain reaction (RT-qPCR) using specific markers, including scleraxis (Scx), tenascin C (TN-C), and tenomodulin (Tnmd) (Additional file 2: Fig. S2B). Cells of passages 2–4 were used for follow-up experiments.

### Establishment of a co-culture model in vitro

To determine the intervention concentration of hydrogen peroxide (H_2_O_2_) on tenocyte apoptosis, a Cell Counting Kit-8 (CCK-8, APExBIO, Houston, USA) was used. Cultured tenocytes were treated with 250 μM H_2_O_2_ for 12 h, which caused a 50%-60% reduction in cell viability (Additional file 3: Fig. S3A and B).

To visualize mitochondrial transfer from MSCs to tenocytes, a MitoTracker dye (Beyotime Biological Technology, Shanghai, China)-based staining assay was used as previously provided [11, 23]. Briefly, the tenocytes were pretreated with 250 μM H_2_O_2_ for 12 h. Subsequently, the mitochondria of injured tenocytes were labeled with a 200 nM MitoTracker Green probe at 37 °C and 5% CO_2_ for 35 min, and then, the nuclei were labeled with Hoechst 33,342 (Beyotime) for 10 min. To distinguish MSCs from tenocytes in mixed cultures, MSC mitochondria were labeled with 200 nM MitoTracker Red CMXRos for 25 min before co-culture. Excess probe dye was washed out with DMEM/F12. Stained MSCs (5 × 10^4^) were added to stained tenocytes at a 1:1 ratio in a six-well plate in DMEM/F12 supplemented with 10% FBS and 1% P/S for 48 h.

To induce mitochondrial dysfunction, MSCs were pretreated with 25 μM rotenone (Rot, a mitochondrial respiratory inhibitor; Macklin Chemical Technology, Shanghai, China) for 2 h, as previously described [30]. Furthermore, the co-culture medium was prepared in the presence or absence of 350 nM cytochalasin B (CB, a blocker of tunneling nanotubes (TNTs) formation to inhibit mitochondrial transfer; Meilunbio Biological Technology, Suzhou, China) [31]. An inverted fluorescence microscope (Olympus IX53, Tokyo, Japan) equipped with an Olympus DP73 digital camera and Olympus cellSens standard software was used to observe mitochondrial transfer after 48 h of co-culture.

### Flow cytometry and cell sorting

To assess the effects on mitochondrial transfer, 1 × 10^7^ tenocytes were labeled with CellTracker™ Violet (CTV, Invitrogen) to distinguish them from MSCs in the co-culture system. CTV-positive cells were sorted using a FACSAria III sorter (BD Biosciences). Following cell sorting, the tenocytes were evaluated for apoptosis, oxidative stress, and mitochondrial function.

Tenocyte proliferation was assessed using flow cytometry (BD FACSCelesta) to determine the number of CTV-positive cells. Apoptosis was detected using an Annexin V-FITC Apoptosis Detection Kit (Beyotime). Briefly, sorted cells were harvested, re-suspended in 195 μL annexin V binding, and stained with 5 μL Annexin V-FITC and 10 μL PI for 10 min at 22–26 °C. A BD FACSCelesta analyzer was used to detect the distribution of cell populations in different quadrants.

### Animal study design

Rodent models of collagenase-induced AT were constructed based on a method previously described, with minor modifications [23, 32]. Forty Sprague–Dawley rats (6–8 weeks old) were randomized using a random number table and divided into the following four groups (*n* = 10): normal control (NC), positive control (AT + Phosphate-Buffered Saline (PBS)), MSC treatment (AT + MSC), and CB interference (AT + MSC + CB). Each rat was anesthetized with halothane (2%). During anesthetization, 0.8 mg/50 μL collagenase type I (Solarbio) was injected into the bilateral Achilles tendon tissues using a microliter syringe (Hamilton, Switzerland). Two weeks of injection caused disorganized arrangements of collagenous fibers, as detected by Picrosirius red (PSR) staining and immunofluorescence assay. BMSCs were pre-labeled with MitoTracker Red CMXRos and re-suspended at a concentration of 1 × 10^7^ cells/mL in PBS. Briefly, each Achilles tendon was treated as follows. The collagenase-induced lesions were injected with 20 μL PBS or BMSCs solution. Meanwhile, 20 μL MSCs containing a solution of CB (final concentration: 350 nM) was introduced into each side of the Achilles tendon by local injection, as the MSC + CB group. The rats of each group were raised in single cages. Animals were maintained in the Laboratory Animal Center of Southeast University. The single animals were acclimatized to laboratory conditions (22–25 °C, 12-h/12-h light/dark, 50% humidity, ad libitum access to food and water). All of the animals survived the experiment period. The animals were killed using carbon dioxide 5 or 14 days post-treatment. Achilles tendons were harvested and evaluated at both time points, whereas the fluorescence assay of mitochondrial transfer was evaluated on day 5 post-treatment only.

### RT-qPCR

RNA was isolated from cells or tendon tissues using TRIzol reagent (Servicebio). RNA concentration and purity were determined using a NanoDrop2000 spectrophotometer (Thermo Fisher). RNA was converted to cDNA using a SweScript RT I First Strand cDNA Synthesis Kit (Servicebio). Briefly, total RNA (2 µg) was incubated with Oligo (dT)_18_ Primer (0.5 μL), Random Hexamer primer (0.5 µL), 5 × Reaction Buffer (4 μL), Servicebio® RT Enzyme Mix (1 μL), and nuclease-free water (20 μL in total). RT-qPCR was performed using 2 × SYBR Green qPCR Master Mix (None ROX) (Servicebio). The forward and reverse primer sequences are listed in Additional file 4: Table S1. Relative quantification of gene expression was performed in the experimental groups compared to the control group using the 2^−ΔΔCT^ method [29]. mRNA levels were normalized to those of GAPDH.

### Western blot

Western blot was conducted as previously described [23]. Briefly, protein concentration in the supernatant of cells was determined using a BCA Protein Assay Kit (Servicebio). Proteins (30 μg) were separated on an SDS-PAGE gel (Beyotime) and transferred to PVDF membranes. Subsequently, the membranes were incubated overnight with primary antibodies against anti-Bcl-2, anti-Bax, anti-caspase 3, anti-caspase 9, anti-Cytochrome c (Cyt-c), anti-apoptosis-inducing factor (AIF), anti-Smac/DIABLO, anti-Tnmd, anti-MMP-1, anti-Col I, anti-Col III, anti-Dynamin-related protein 1 (Drp1), anti-Mitofusin 2 (Mfn2), and anti-β-actin. The membrane was then incubated with horseradish peroxidase (HRP)-conjugated secondary antibody for 1 h at 22–26 °C. Protein bands were visualized using the BeyoECL Moon chemiluminescence system (Beyotime). All antibody information and dilutions are listed in Additional file 5: Table S2.

### Mitochondrial membrane potential measurement

ΔΨm was measured by JC-1 staining (Beyotime). Tenocytes were rinsed with PBS and incubated with 5 μM JC-1 dye at 37 °C for 20 min. Cell nuclei were stained with Hoechst 33,342 (Beyotime) for 10 min. Images were captured using a confocal microscope (Olympus FluoView FV3000; Japan) with a 100 × oil-immersion objective lens (1.40 NA; Olympus) and captured with FluoView software (FV31S-SW, Olympus). The ratio (%) of the red/green fluorescence intensity was calculated relative to that of the control group.

### Adenosine triphosphate measurement

ATP content was detected using an ATP Determination Kit (Invitrogen). Briefly, a reaction buffer solution containing D-luciferin, firefly luciferase, and DTT was freshly prepared, and 10 µL of the cell lysate was mixed with 90 µL of the reaction buffer. An ATP standard solution or sample containing ATP was added to each well, and luminescence was detected at 560 nm using an FLx800TM Fluorescence microplate reader (BioTek, USA). The ATP concentration was determined from the standard curve.

### Evaluation of mitochondrial permeability transition pore (mPTP)

mPTP of sorted tenocytes was analyzed using the mPTP Assay Kit (Beyotime) as previously described [33]. The relative fluorescence intensity (RFI) was measured using flow cytometry. If the RFI decreased, the openness of mPTP increased.

### Oxygen consumption rate (OCR) measurement

To assess mitochondrial bioenergetics, OCR was measured as previously described [34]. Sorted tenocytes (5 × 10^4^ cells/well) were plated on a Seahorse XFe 24 microplate extracellular flux analyzer (Seahorse Biosciences, USA). Cells were incubated for 1 h at 37 °C in XF base medium (Seahorse Biosciences) containing 11 mM glucose (Sigma), 2 mM glutamine (Sigma), 1 mM pyruvate (Sigma), 2 μM oligomycin (Sigma), 4 μM FCCP, and 2 μM rotenone/antimycin A (Macklin/Sigma). The Seahorse XF24 Analyzer was run using 8-min cyclic protocol commands (mix for 3 min, let stand for 2 min, and measure for 3 min). The results were normalized to the total protein content in each well.

### Reactive oxygen species (ROS) measurement

Mitochondrial ROS (mito-ROS) levels were analyzed by MitoSOX™ Red (Invitrogen). Tenocytes were stained with MitoSOX (5 μM) for 10 min at 37 °C and analyzed by BD FACSCelesta. In addition, the level of intracellular ROS (intra-ROS) was detected using 2′,7′-dichlorofluorescin diacetate (DCFH-DA) probe (Beyotime). Tenocytes were stained with DCFH-DA probe (10 μM) for 20 min at 37 °C. Cell nuclei were counterstained with Hoechst 33,342 (Beyotime) for 10 min. DCF fluorescence images were captured using a confocal microscope (Olympus FluoView FV3000; Japan) with a 100 × oil-immersion objective lens (1.40 NA; Olympus) and captured with FluoView software (FV31S-SW, Olympus). The DCF fluorescence intensity was normalized to that in normal control tenocytes.

### Enzyme-linked immunosorbent assay (ELISA)

Concentrations of interleukin (IL) -6 and IL-1β in the supernatant of cell co-culture medium or tendon tissue were measured using an IL-6 ELISA kit (Thermo Fisher) and IL-1β ELISA kit (Multi Sciences, Hangzhou, China), respectively. Briefly, the supernatant samples were added to the wells in triplicate and incubated for 2 h at 37 °C. Then, they were incubated in an HRP conjugate solution for 30 min at 37 °C and TMB solution for 30 min at 22–26 °C. ELISA plates were detected at 450 nm using an FLx800TM Fluorescence microplate reader (BioTek, USA). Quantitative data are presented as average concentrations in pg/mL.

### Assessment of mitochondrial transfer in vivo

To visualize the distribution of the labeled mitochondria after MSC injection, Achilles tendon tissues were harvested and cryosectioned as previously mentioned [23]. Briefly, the samples were embedded in an OCT compound and the tendon tissue was cut to a thickness of 5 µm. The attached sections were stained with 300 nM MitoTracker Green (Beyotime) at 37 °C for 30 min. Subsequently, the cells were stained with Hoechst 33,342 (Beyotime). Representative images were acquired using a fluorescence microscope (Olympus IX53).

### Histological analysis

Five and 14 days post-treatment, bilateral Achilles tendon tissues were harvested and paraffin-embedded, and fixed specimens were sectioned at a thickness of 5 µm, according to the standard procedure. Subsequently, the slices were stained with hematoxylin and eosin (H&E), Masson’s trichrome, and Picrosirius red (PSR). Immunohistochemistry (IHC) was performed using CD68 (a tissue macrophage marker) and matrix metalloproteinase 9 (MMP-9). The distribution of Col I and Col III was evaluated by immunofluorescence staining, using Servicebio Technology (Wuhan, China). All antibody information and dilutions are listed in Additional file 5: Table S2. Sections were examined and photographed using a light or fluorescence microscope (BX53 or IX53, Olympus, Japan).

### Statistical analysis

Statistical analyses were performed using SPSS version 26 and GraphPad Prism version 9. Significant differences were analyzed by unpaired Student's *t*-test for comparisons between two groups and one-way analysis of variance (ANOVA) (Bonferroni or LSD method) for multiple-group comparisons. Quantitative results are presented as mean ± standard deviation (SD). The statistical value of *P* < 0.05 was significant.

## Results

### Mitochondrial transfer from MSCs to injured tenocytes triggers the anti-apoptotic functions of MSCs

**Fig. 1:**
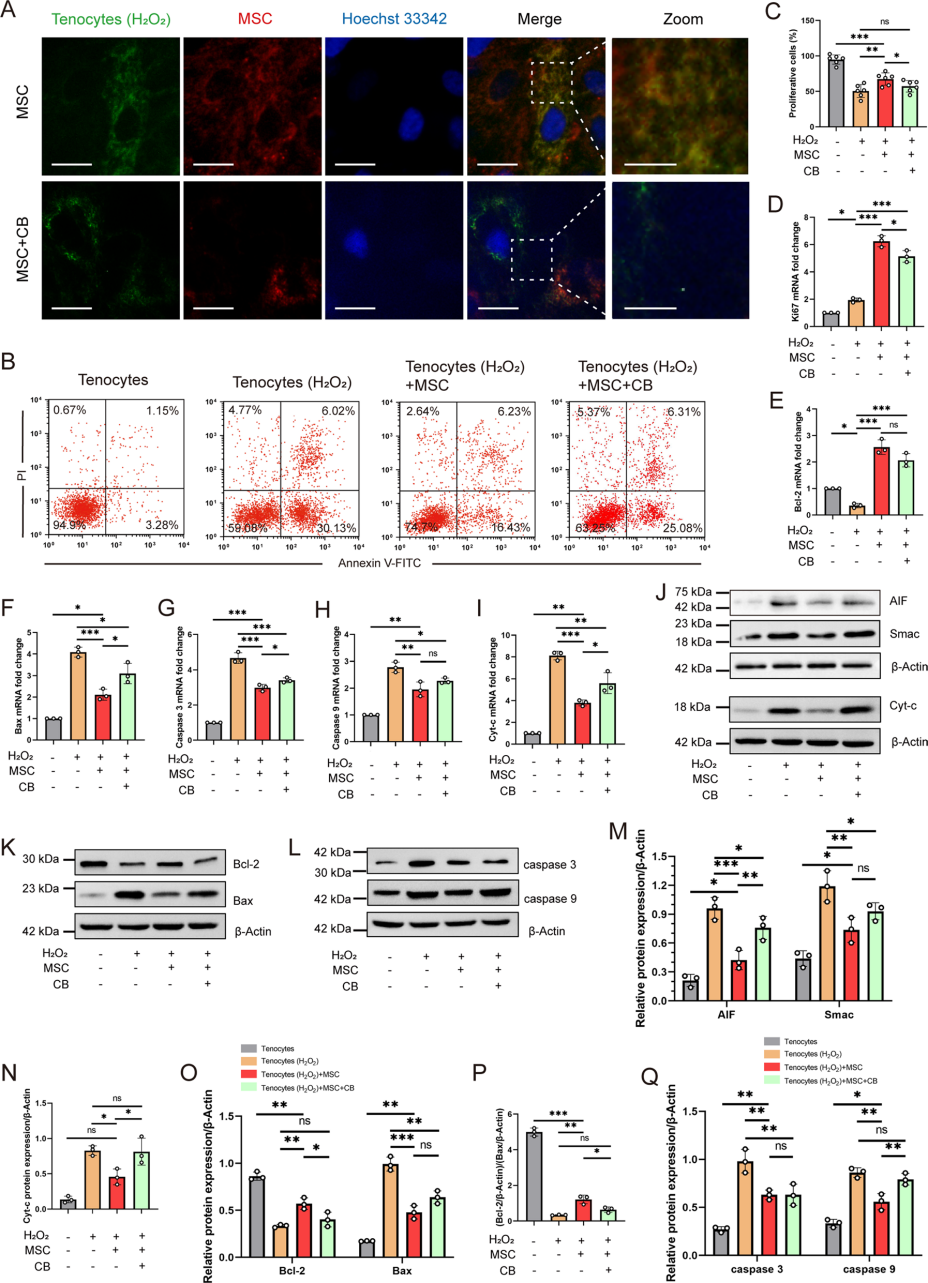
Mitochondria of MSCs successfully transferred to H_2_O_2_-induced tenocytes alleviate in vitro apoptosis. **A** Representative fluorescence images showing MitoTracker Red CMXRos-labeled MSC mitochondria (red) in tenocytes (green). Nuclei are counterstained with Hoechst 33,342 (blue). Scale bars: 200 × , 25 µm; 400 × , 10 μm (magnified graphs). **B** Flow cytometry results displaying annexin V-/PI- (viable), annexin V + /PI- (early apoptotic), annexin V + /PI + (late apoptotic) , or annexin V-/PI + (necrotic) tenocytes (*n* = 3). **C** Tenocyte proliferation was determined using CellTrace™ Violet by flow cytometry (*n* = 6). **D** Gene expression of proliferation factor Ki67. The relative gene expression level (Gene/GAPDH relative to the control) was measured by RT-qPCR (*n* = 3). MSC treatment resulted in an increase of anti-apoptotic gene Bcl-2 (**E**), and decrease of pro-apoptotic genes, including Bax (**F**), caspase 3 (**G**), caspase 9 (**H**), and Cyt-c (**I**), in H_2_O_2_-injured tenocytes (E-I, *n* = 3). Mitochondrial transfer prevented H_2_O_2_-induced mitochondrial apoptosis gene expression, compared to the mitochondrial transfer interference by CB. Bcl-2 protein expression (protein/β-actin relative to control) (**K, O**) was shown by western blot analysis to be significantly upregulated in MSC group, whereas expression of AIF (**J, M**), Smac/DIABLO (**J, M**), Cyt-c (**J, N**), Bax (**K, O**), caspase 3 (**L, Q**), and caspase 9 (**L, Q**) were downregulated, compared to H_2_O_2_-treated tenocytes (J-L, *n* = 3). **P** Expression rate of Bcl-2 and Bax. Data represent mean ± SD. Statistical significance of the differences among groups was determined by ANOVA (Bonferroni or LSD method). ^*^P < 0.05, ^**^P < 0.01, ^***^P < 0.001

To determine whether mitochondrial transfer from MSCs to H_2_O_2_-induced tenocytes occurred, we co-cultured MSCs with tenocytes. The mitochondria of MSCs and tenocytes were stained with MitoTracker Red CMXRos and MitoTracker Green fluorescent dye, respectively. The nuclei of the tenocytes were stained with Hoechst 33,342. Mitochondria with fluorescent dyes were successfully built to reveal mitochondrial transfer from MSCs to H_2_O_2_-induced tenocytes (Fig. 1A and Additional file 6: Fig. S4). We further investigated the mechanism by which CB inhibits the formation of tunneling nanotubes (TNTs) between donor and recipient cells to block mitochondrial transfer. We found CB reduced mitochondrial transfer from MSCs to injured tenocytes (Fig. 1A and Additional file 6: Fig. S4). To determine whether mitochondrial transfer protects against H_2_O_2_-induced cytotoxicity, injured tenocytes were treated with MSCs. Annexin V-FITC and PI staining revealed early apoptotic (annexin V + /PI-) and late apoptotic (annexin V + /PI +) tenocytes. The percentage of early apoptotic tenocyte populations after MSC treatment was decreased compared to that in the H_2_O_2_-induced tenocyte and CB intervention groups (Fig. 1B). The tenocytes were labeled with CellTrace Violet, and flow cytometry was used to measure the number of CTV-positive tenocytes. The results showed that MSCs significantly promoted the proliferation of injured tenocytes (Fig. 1C). In addition, we assessed Ki67 mRNA expression, a marker of cell proliferation [35]. RT-qPCR revealed that Ki67 expression increased markedly in the MSC-treated group, whereas tenocytes exposed to H_2_O_2_ exhibited low Ki67 levels (3.2-fold vs. MSC-treated, *P* < 0.001) (Fig. 1D). The blocked mitochondrial transfer by CB demonstrated the Ki67 level was 0.8 times higher than that in the MSC group (*P* = 0.013).

Activation of the mitochondria-mediated apoptotic pathway is induced by oxidative stress and depolarization of ΔΨm in response to H_2_O_2_ treatment [29]. We measured the expression of pro-apoptotic and anti-apoptotic genes at the mRNA and protein levels (Fig. 1E–Q). Tenocytes exposed to 250 μM H_2_O_2_ displayed higher levels of pro-apoptotic markers (caspase 3, caspase 9, Cyt-c, and Bax) and lower levels of anti-apoptotic gene/protein (Bcl-2) than the control group. RT-qPCR and western blot analysis further confirmed that mitochondrial transfer by MSCs significantly decreased caspase 3, caspase 9, Cyt-c, and Bax levels and increased Bcl-2 mRNA and/or protein levels, compared to the CB-treated groups. Likewise, western blot analysis indicated that mitochondrial transfer from MSCs significantly decreased the functional mitochondrial proteins (Cyt-c, AIF and Smac/DIABLO) levels in tenocytes, compared to the CB-treated groups (Fig. 1J, M and N). Notably, the expression level of Bcl-2/Bax in the MSC group was 1 time higher than in the CB-treated group (*P* = 0.03) (Fig. 1P). Together, these findings demonstrate that mitochondrial transfer rescued injured tenocytes from apoptosis in vitro, and this anti-apoptotic effect can be partially blocked by CB, suggesting mitochondrial transfer as a potential mechanism in MSC therapy.

### MSC-derived mitochondria rescued mitochondrial function from injured tenocytes

**Fig. 2:**
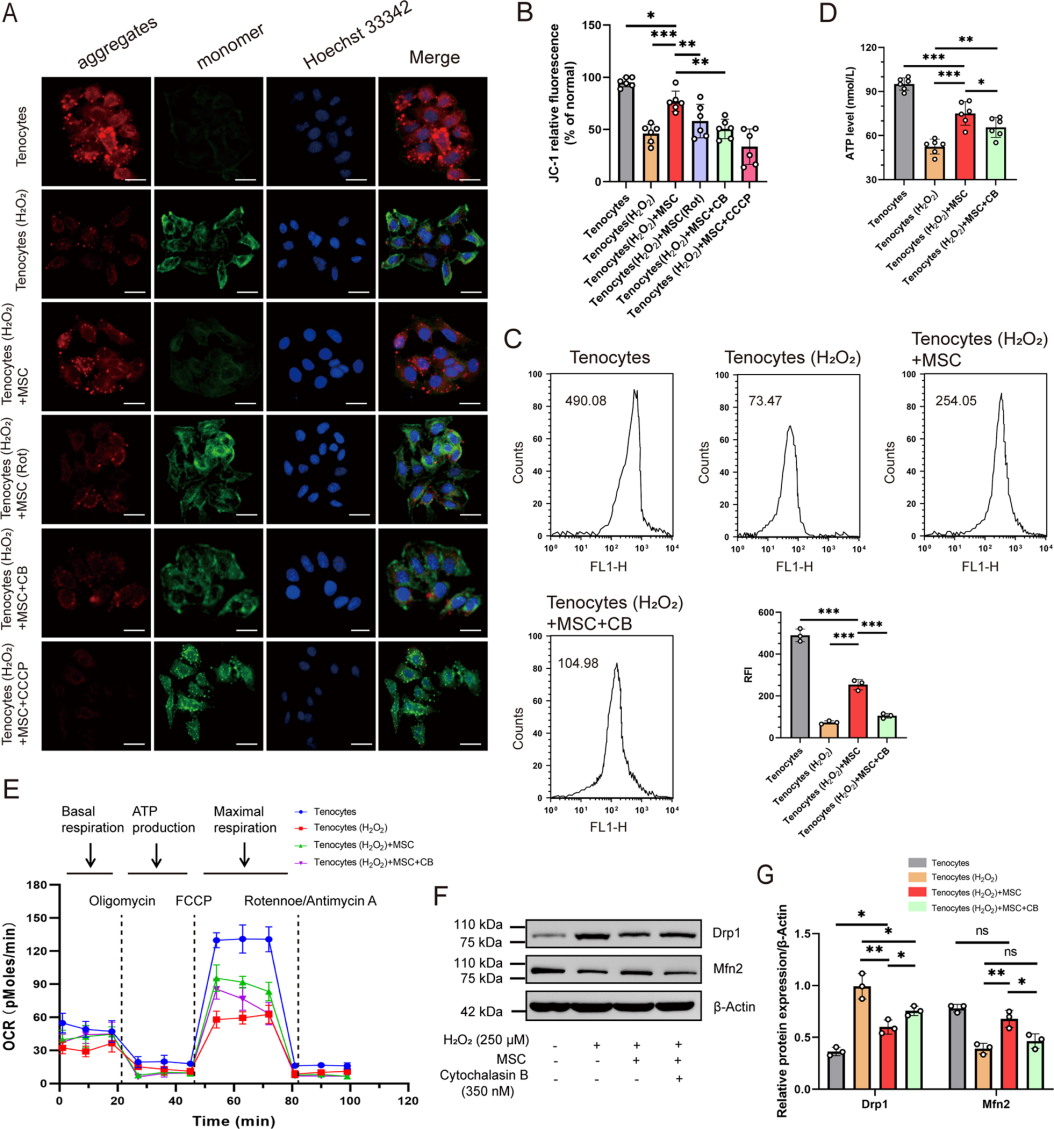
Functional measurements of mitochondrial activity in rescued tenocytes. **A** ΔΨm was determined by confocal microscopy using JC-1 dye (*n* = 6). JC-1 aggregates show red and JC-1 monomers green fluorescence. The nuclei were stained with Hoechst 33,342 (blue). Scale bar: 400 × , 25 µm. **B** Fluorescence quantitative analysis of ΔΨm for Fig. 2A. **C** Representative graphs illustrate the decrease in mPTP opening after MSC treatment. Quantitative mPTP in tenocytes is represented by RFI (*n* = 3). **D** Quantitative analysis of ATP using the ATP determination kit (*n* = 6). **E** OCR were determined by Seahorse XFe 24 Analyzer and normalized to protein content (*n* = 6). **F** Representative western blots of total protein expression of Drp1 and Mfn2 (*n* = 3). **G** Histogram analysis showing the relative protein levels for Fig. 2F. Data represent mean ± SD. Statistical significance of the differences among groups was determined by ANOVA (Bonferroni or LSD method). ^*^P < 0.05, ^**^P < 0.01, ^***^P < 0.001

Diverse functional assessments were performed on mitochondria isolated from rescued tenocytes. ΔΨm was measured using the JC-1 probe. The H_2_O_2_-treated group showed an approximately 48% reduction in ΔΨm (vs. without exposure to H_2_O_2_, *P* < 0.001). However, ΔΨm increased to that in the MSC group (1.7-fold vs. H_2_O_2_ exposure, *P* < 0.001) (Fig. 2A and B). The potential of MSCs to recover ΔΨm was reduced by pre-treatment of MSC with Rot or CB in co-culture. The opening of mPTP is associated with the loss of ΔΨm and is considered an initial step in activation apoptosis [33]. The RFI value increased significantly after MSC treatment, implying that the mPTP opening rate in the MSC group was reduced (Fig. 2C). ATP levels were measured using an ATP determination kit. Cultured tenocytes treated with MSCs displayed higher ATP levels than those in the MSC + CB group (*P* = 0.02) (Fig. 2D). The mitochondrial OCR was then measured using Seahorse XF24. The injured tenocytes treated with MSCs showed a marked increase in basal respiration, ATP production, and maximal respiration compared to the H_2_O_2_-treated or MSC + CB groups (Fig. 2E). The balance between mitochondrial fusion and fission is disrupted by mitochondrial membrane depolarization [36], and we measured the levels of the mitochondrial fission factor (Drp1) and fusion factor (Mfn2) by western blot analysis. We observed a decrease of approximately 60.6% in Drp1 levels, whereas Mfn2 levels increased approximately 1.7 times in the MSC-treated group compared to the H_2_O_2_-treated group (both *P* < 0.01) (Fig. 2F and G). However, blocking mitochondrial transfer with CB significantly reduced this effect (both *P* < 0.05). These results illustrate that mitochondrial transfer can contribute to the restoration of mitochondrial function in injured tenocytes by improving ΔΨm and ATP production.

### Mitochondrial transfer improved Tnmd and Col I expression and decreased MMP-1 and Col III expression in H_2_O_2_-induced tenocytes

**Fig. 3:**
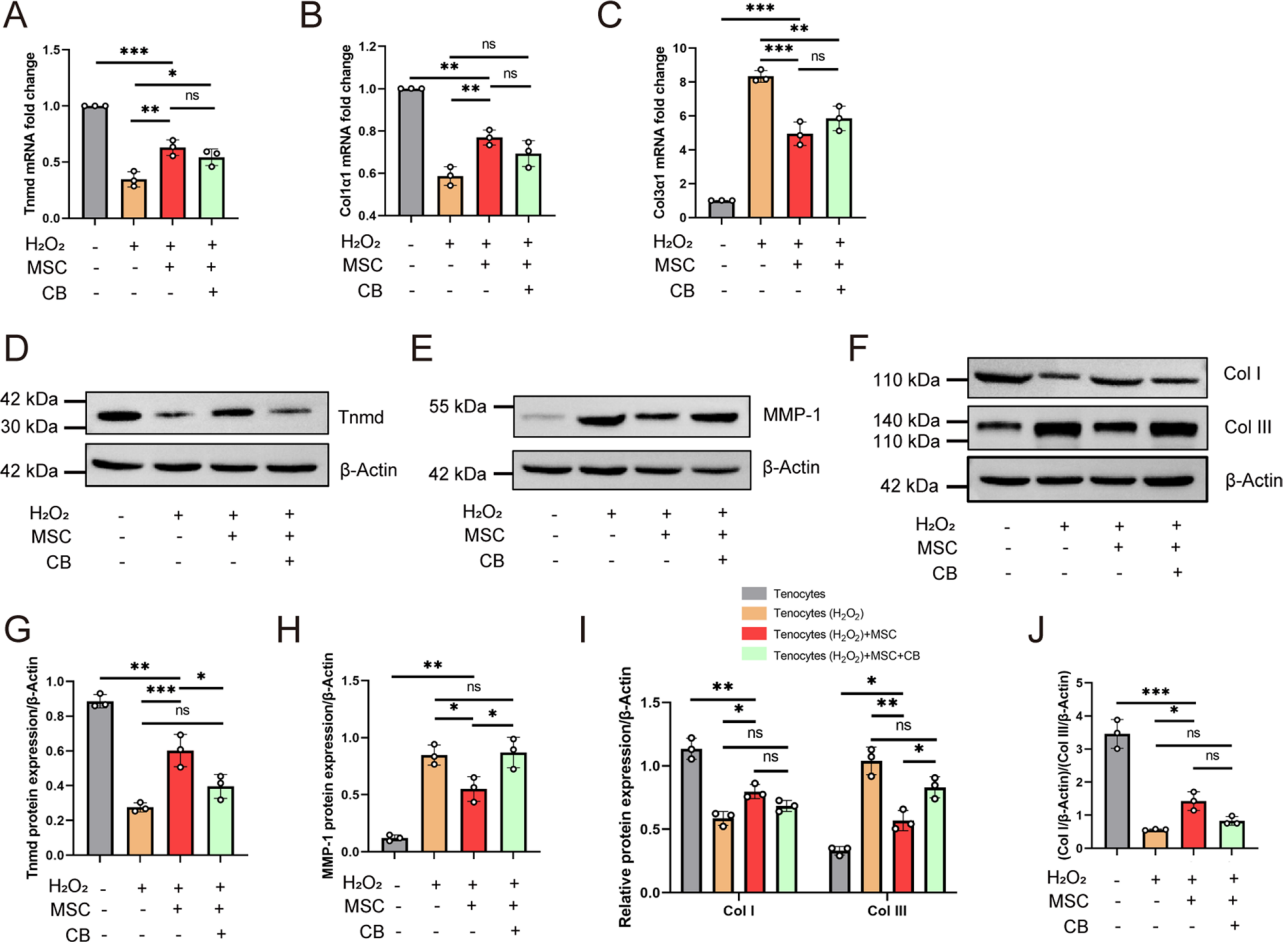
Mitochondrial transfer promotes collagen fibril formation and inhibits collagen fibril degradation. RT-qPCR was conducted to detect tenocyte-lineage marker levels (**A**, Tnmd; **B**, Col1α1; **C**, Col3α1) (*n* = 3). Protein expression of Tnmd (**D**), MMP-1 (**E,** degrade collagen fibrils), Col I (**F**), and Col III (**F**) was determined by western blot (*n* = 3). **G-I** Densitometric analysis of the western blot shown in Fig. 4D–F. **J** Histogram represents the Col I/Col III ratio. Data represent mean ± SD. Statistical significance of the differences among groups was determined by ANOVA (Bonferroni method). ^*^P < 0.05, ^**^P < 0.01, ^***^P < 0.001

RT-qPCR and western blot were used to investigate the expression of tenocyte-related markers (Tnmd, Col I and Col III) and MMP-1 (degrade collagen fibrils) [23, 37]. The expression of Tnmd decreased approximately 2.8 times after exposure to H_2_O_2_ (vs. without exposure to H_2_O_2_,* P* < 0.001) and recovered 48 h after MSC treatment (vs. exposure to H_2_O_2_,* P* = 0.003) (Fig. 3A). Next, we investigated whether mitochondrial transfer might affect differential collagen synthesis in vitro, because Col III expression is a predominant feature of tendinopathy [38], compared to Col I expression. Col1α1 expression was also suppressed approximately 1.7 times in injured tenocytes (vs. without exposure to H_2_O_2_,* P* < 0.001) and increased in the MSC-treated group (vs. exposure to H_2_O_2_,* P* = 0.004) (Fig. 3B). However, Col3α1 expression increased by approximately 8.3 times (vs. without exposure to H_2_O_2_,* P* < 0.001) and decreased in the MSC-treated group (vs. exposure to H_2_O_2_,* P* < 0.001) (Fig. 3C). Interestingly, CB treatment showed a trend toward decreased Tnmd and Col1α1 levels, but elevated Col3α1 levels, although this was not statistically significant in the MSC group.

Western blot analysis demonstrated similar results at the protein level. Tnmd levels in the MSC group increased approximately 2.2 times (vs. exposure to H_2_O_2_,* P* < 0.001) and decreased approximately 0.6 times in the CB intervention group (vs. MSC group,* P* = 0.022) (Fig. 3D and G). The level of MMP-1, a collagenase that degrades collagen fibers for matrix regulation [39, 40], increased approximately 7 times in injured tenocytes (vs. without exposure to H_2_O_2_,* P* < 0.001) and decreased after mitochondrial transfer (MSC-treated vs. H_2_O_2_ exposure,* P* = 0.035) (Fig. 3E and H). Expression of Col I, the major component of the tendon matrix, downregulated compared to that in the no H_2_O_2_ exposure group and was upregulated after mitochondrial transfer (Fig. 3F and I). In contrast, Col III protein levels showed an opposite trend. Compared to the H_2_O_2_ group, the Col I/Col III ratio was significantly elevated in the MSC group (*P* = 0.024) (Fig. 3J). Notably, mitochondrial transfer partly blocked by CB trended toward decreased Tnmd and Col I levels, but increased MMP-1 and Col III levels. Together, these results indicate that mitochondrial transfer remodels the tendon structure and represses collagen degradation.

### Mitochondrial transfer attenuates H_2_O_2_-induced oxidative stress and inflammation in cultured tenocytes

**Fig. 4:**
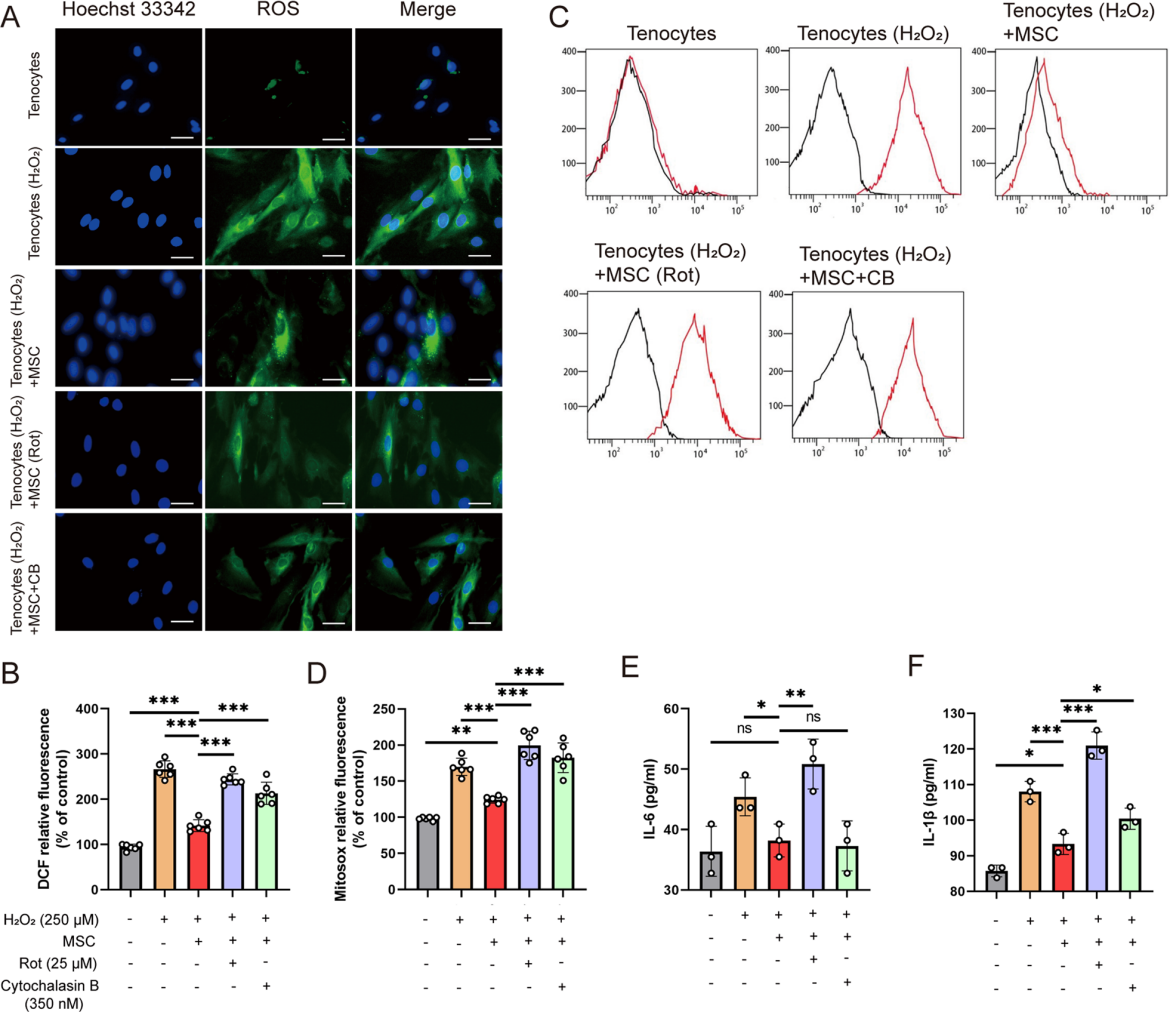
Mitochondrial transplantation reduces elevated levels of oxidative stress and inflammation. **A** Intracellular ROS (green) levels were determined by DCF-DA staining (*n* = 6). The nuclei were stained with Hoechst 33,342 (blue). Scale bar: 400 × , 25 µm. **B** Histograms represent intracellular ROS quantification for Fig. 4A. **C** Mitochondrial ROS levels were assessed by flow cytometry with MitoSOX staining (*n* = 6). **D** Histograms represent mitochondrial ROS quantification for Fig. 4C. Supernatant inflammation-related markers, such as IL-6 (**E**) and IL-1β (**F**), were detected by ELISA (*n* = 3). Data represent mean ± SD. Statistical significance of the differences among groups was determined by ANOVA (Bonferroni or LSD method). ^*^P < 0.05, ^**^P < 0.01, ^***^P < 0.001

To determine the effect of mitochondrial transfer on oxidative stress, we measured ROS generation using DCF-DA (intra-ROS) and MitoSOX Red (mito-ROS) probe assays. Mitochondrion is a major source of ROS, and H_2_O_2_ disruption increased both intra- and mito-ROS production (Fig. 4A–D). A sustained decrease in intra-ROS and mito-ROS levels was observed in H_2_O_2_-induced tenocytes after MSC treatment (H_2_O_2_ exposure vs. MSC group, both* P* < 0.001) (Fig. 4A–D). Mitochondrial transfer suppressed intra-ROS (33.3%) and mito-ROS (31.9%) in MSC treatment versus exposure to CB-inhibited mitochondrial transfer (both *P* < 0.001) (Fig. 4B and D). Specifically, we measured the levels of pro-inflammatory cytokines (IL-6 and IL-1β) [41] using ELISA. The results showed that MSCs significantly decreased IL-1β and IL-6 levels in tenocytes compared to those of the only H_2_O_2_ exposure group (IL-6: 38.20 ± 2.71 vs. 47.23 ± 3.13, *P* = 0.014; IL-1β: 93.30 ± 2.96 vs. 108.04 ± 2.86, *P* < 0.001) (Fig. 4E and F). The decreasing trend in IL-6 and IL-1β levels in the mitochondrial transfer group (MSC-treated) was weakened by CB involvement. We also investigated whether oxidative stress could be generated in vitro by inducing mitochondrial damage in MSCs pre-exposed to rotenone. Figure 4A–F reveals that rotenone-induced mitochondrial damage in MSCs did not decrease the elevated level of oxidative stress and inflammation, indirectly indicating that transferring damaged or dysfunctional mitochondria in co-culture affected those of tenocytes.

### Mitochondrial transfer stimulated Achilles tendinopathy healing efficacy of MSCs in vivo

**Fig. 5:**
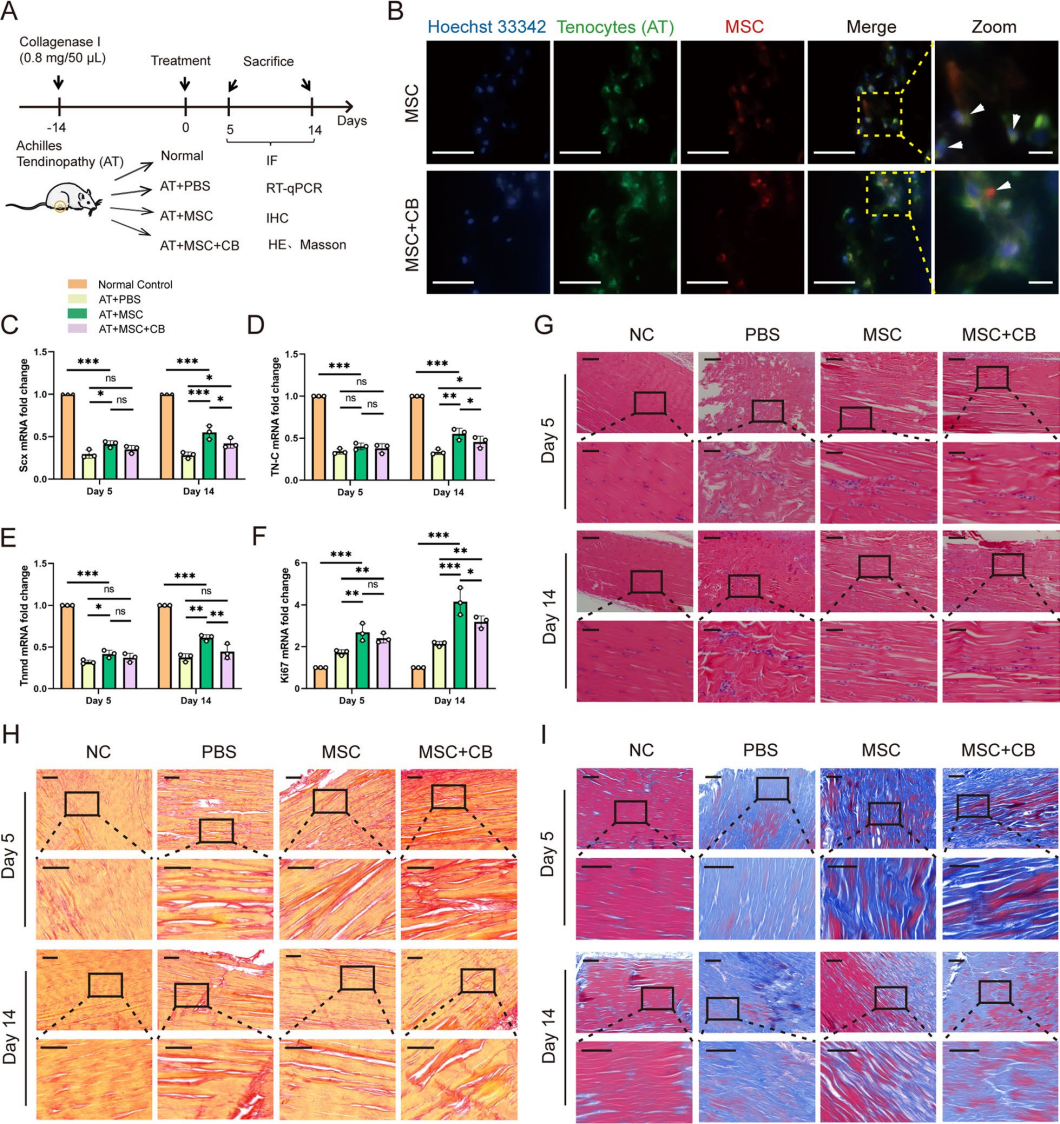
Mitochondrial transfer improves the severity of collagenase I-induced Achilles tendinopathy in rats. **A** In vivo experimental protocol. **B** Representative image of the tendon tissue after injection of MSC: red and green indicate MSC mitochondria (MitoTracker Red CMXRos) and harvested tendon tissue mitochondria (MitoTracker Green), respectively, on day 5 after collagenase injection with DAPI nuclear staining (blue). CB was used to block mitochondrial transfer. Mitochondria of MSCs (white arrowheads) localized in tenocytes within the tissue. Scale bars: 100 × , 25 µm; 400 × , 5 μm (magnified graphs). Relative Scx (**C**), TN-C (**D**), and Tnmd (**E**) mRNA levels in tendon tissue assessed by RT-qPCR (C-E, *n* = 3). **F** Level of proliferation factor Ki67 assessed by RT-qPCR (*n* = 3). **G** H&E staining of the tendons in each group (*n* = 3). Scale bars: 100 × , 200 μm; 400 × , 50 μm (magnified graphs). **H** Collagen was evaluated with PSR staining (*n* = 3). Scale bars: 100 × , 100 μm; 200 × , 50 μm (magnified graphs). **I** Masson staining (*n* = 3)*.* Blue indicates collagen matrix disruption, whereas red indicates collagen fibers. Scale bars: 100 × , 100 μm; 200 × , 50 μm (magnified graphs). Data represent means ± SD. Statistical significance of the differences among groups was determined by ANOVA (LSD method). ^*^P < 0.05, ^**^P < 0.01, ^***^P < 0.001

To evaluate the therapeutic efficacy of MSCs in mitochondrial transfer in functional recovery, a rat model of tendinopathy was studied by injecting collagenase type I into the Achilles tendon [23] and randomly assigned to four groups (Fig. 5A). We evaluated whether the injected MitoTracker Red CMXRos-labeled MSCs were incorporated into the Achilles tendon. MitoTracker Red CMXRos signals were detected in harvested tendon tissues after 5 days. A greater number of red signals were detected in the Achilles tendon in MSC-treated rats than in MSC + CB-treated rats (Fig. 5B). The relative expression of tendon-specific markers (Scx, TN-C, and Tnmd) determined on day 14 after treatment was significantly higher in rats treated with MSC than in rats who received either PBS or CB (Fig. 5C–E). However, the differences in the above expressions were not statistically significant on day 5. The proliferation marker mRNA expression (Ki67 mRNA) was then assessed after treatment (Fig. 5F). Ki67 expression increased dramatically by approximately 1.5 times on day 5 and 1.9 times on day 14 in the group treated with MSC, compared to that in the PBS-injected tendon tissue (both *P* < 0.01).

To examine tendon organization, Achilles tendon tissues were analyzed by staining with H&E, Picrosirius red, and Masson’s trichrome. H&E-stained images (Fig. 5G) demonstrated increased cellularity in the PBS, MSC, and MSC + CB groups. In particular, the tissues of the MSC group on day 14 revealed a markedly denser cell population than the MSC + CB group. The PSR-stained images (Fig. 5H) demonstrated the separation of the fiber bundles and the loss of the normal fiber demarcation pattern in the PBS group. An improvement in collagen organization was observed on days 5 and 14 in both the MSC and MSC + CB groups. Interestingly, collagen fibers were arranged more neatly in the MSC group than in the MSC + CB group. Furthermore, to evaluate whether mitochondrial transfer effectively prevented collagen matrix disruption, tendon tissues were stained with Masson’s trichrome (Fig. 5I). Consistent with previous results [32], the highly organized collagen fibril structures in normal tendons were severely disrupted by injection of collagenase I. MSCs were more effective in preventing collagen disruption and disorganization than MSC + CB treatment. Together, the results presented above imply that MSCs contribute to tendon remodeling by mitochondrial transfer.

### Mitochondrial transfer inhibited inflammation and ameliorated tendon extracellular matrix composition in vivo

**Fig. 6:**
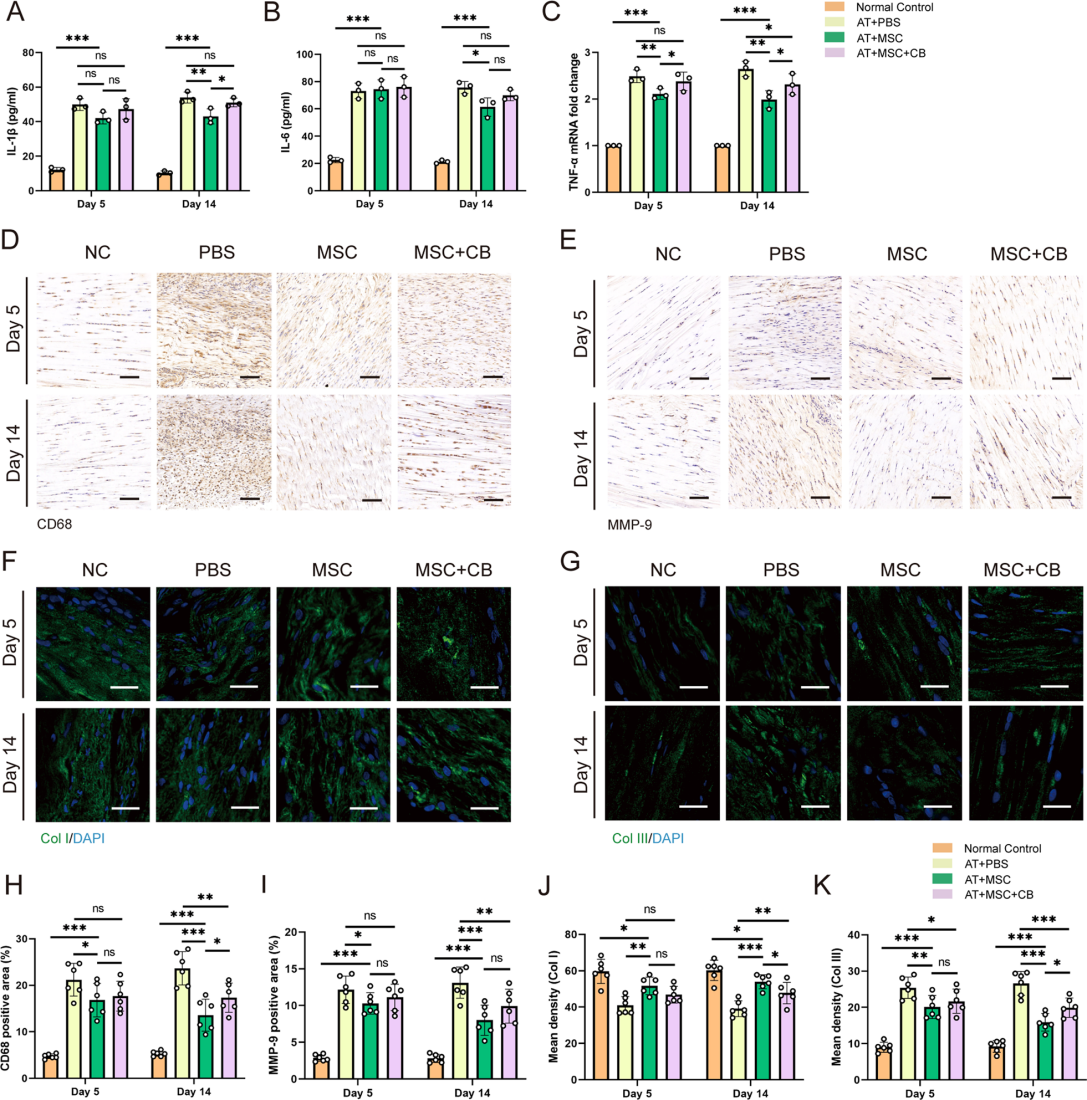
Impact of mitochondrial transfer on inflammatory and tendon matrix-related factors. The pro-inflammatory factors IL-1β (**A**) and IL-6 (**B**) were counted by ELISA in tendon tissues from each group (*n* = 3). **C** Level of pro-inflammatory factor TNF-α was assessed by RT-qPCR (*n* = 3). IHC staining of macrophage-specific CD68 (**D**) and MMP-9 (**E**) (D, E, *n* = 6). D and E, scale bars: 200 × , 100 µm. Quantification of CD68 (**H**) and MMP-9 (**I**) levels for Fig. 6D and E, respectively. IF staining against Col I (**F**) and Col III (**G**) (F, G, *n* = 6). F and G, scale bars: 200 × , 25 µm. **J, K** Quantification analysis of Fig. 6F and G, respectively. Data represent mean ± SD. Statistical significance of the differences among groups was determined by ANOVA (Bonferroni or LSD method). ^*^P < 0.05, ^**^P < 0.01, ^***^P < 0.001

To evaluate the impact of mitochondrial transfer on inflammatory cytokines in tendon tissue, TNF-α, IL-1β, and IL-6 levels were evaluated by ELISA or RT-qPCR on days 5 and 14 after collagenase I injection. Compared to the NC group, IL-1β, IL-6, and TNF-α levels increased approximately 4.5 times (Fig. 6A), 3.3 times (Fig. 6B), and 2.5 times (Fig. 6C), respectively, in the PBS group on days 5 and 14. Furthermore, compared to the CB-inhibited mitochondrial transfer group, mitochondrial transfer significantly impaired collagenase I-induced upregulation of IL-1β or TNF-α expression (Fig. 6A and C). Furthermore, we examined the infiltration of CD68-positive macrophages into the tendon tissues (Fig. 6D and H). On days 5 and 14, the MSC group exhibited a lower macrophage density than the PBS group (16.81 ± 3.61 vs. 21.19 ± 3.52, *P* = 0.020; 13.59 ± 3.44 vs. 23.67 ± 3.58, *P* < 0.001, respectively). On day 14 after treatment, the MSC group also showed a significantly lower CD68 density than the CB group (13.59 ± 3.44 vs. 17.33 ± 3.13, *P* = 0.040).

Matrix metalloproteinases are zinc-dependent endopeptidases that degrade the extracellular matrix [42]. MMP-9 was upregulated following the application of collagenase I and significantly downregulated in the MSC-treated group (Fig. 6E and I). MMP-9 in the MSC group also showed a smaller positive area than in the CB group on days 5 and 14, although the difference was not statistically significant. Consistent with a previous study that reported a reduced expression of Col I in tendinopathy [29], immunofluorescence staining revealed that, compared to normal Achilles tendons, collagenase I treatment significantly decreased Col I level. Compared to the PBS group, MSC treatment significantly increased Col I and decreased Col III levels on days 5 and 14 (Fig. 6F, G, J, and K). Notably, Col I expression increased and Col III decreased in the MSC treatment group compared to the CB-inhibited mitochondrial transfer group (53.95 ± 3.87 vs. 47.74 ± 5.88, *P* = 0.044; 15.88 ± 2.42 vs. 19.89 ± 2.68, *P* = 0.014, respectively) on day 14 after AT established (Fig. 6J and K). Taken together, these results suggest that mitochondrial transfer attenuates the pro-inflammatory response and enhances extracellular matrix deposits.

## Discussion

The findings presented here support the hypothesis that BMSCs donate healthy mitochondria to H_2_O_2_-injured tenocytes in vitro and collagenase-induced Achilles tendon tissues in vivo. This results in the rescue of aerobic respiration, protection of tenocytes from apoptosis, and prevention of Achilles tendinopathy in rats.

Although the effectiveness of MSCs in AT models has been reported, their potential mechanisms remain poorly understood. The proposed mechanisms include MSC differentiation to replace damaged cells and immune regulation effects through the release of paracrine factors or exosomes [11–13]. Mitochondrial transfer from BMSCs to damaged lung epithelial cells as a mechanism of protection against LPS-induced acute lung injury was described by Islam and colleagues (2012) [14], revealing a novel mechanism of MSC-based therapy. Another study confirmed mitochondrial transfer from induced pluripotent stem cell-derived MSCs to airway smooth muscle cells [18]. In our study, mitochondrial transfer from BMSCs to injured tenocytes was observed after 12 h of exposure to H_2_O_2_ and to the Achilles tendon after collagenase induction for 2 weeks. Notably, the fluorescence intensity of the MitoTracker Red CMXRos probe decreased with time after intra-tissue injection (approximately twofold to tenfold decline). Therefore, mitochondrial fluorescence localization was not analyzed on day 14 due to the difficulty in detecting the red fluorescence signal.

In 2004, Rustom et al. [43] first described the novel biological principle of organelle transfer based on TNTs. While gap junctions and cell fusion are also associated with mitochondrial transfer, TNT formation is the most likely contributing mechanism [31]. Furthermore, the abolition of TNT formation by cytochalasin B almost completely blocked mitochondrial transfer [31, 44]. Consistent with these results, mitochondrial transfer was reduced by cytochalasin B, suggesting that mitochondrial transfer was largely mediated by TNTs. Furthermore, the therapeutic effect of MSCs was reduced after mitochondrial transfer was blocked, affecting cell viability, mitochondrial function, and tendon healing.

Interestingly, extensive evidence has shown that rotenone treatment abrogated mitochondrial respiration of MSC but neither improved target cell function nor protected tissue effects [30, 45]. Our results are like these; MSC pretreated with rotenone did not increase ΔΨm or reduce ROS production in target cells, indicating that healthy mitochondria were received to restore recipient cell function. These data are like those previously reported by Lee et al. [23], suggesting that viable and healthy mitochondria are essential for improving mitochondrial function.

We demonstrated that mitochondrial trafficking from BMSCs to tenocytes orchestrates plasticity in the cell and extracellular matrix (ECM). Scx, Tnmd, and TN-C are tendon-specific markers for remodeling [29]. We evaluated the positive effects of MSC treatment on tendon repair ability. Mature tendons consist predominantly of Col I (> 95%), but also comprise minor amounts of Col III [1, 46]. A diseased tendon typically exhibits an increased proportion of Col III relative to Col I [47]. Compared to the PBS group, Col I increased and Col III decreased after MSC intervention on days 5 and 14, respectively. However, there were no statistically significant differences between the MSC and CB groups on day 5. A reason for this was the shorter observation time, as a significant difference was observed on day 14. Treatment with MSCs led to significant improvements in gene/protein expression of Col I and Col III compared to the H_2_O_2_ exposure group in vitro, although no statistically significant improvements were observed in the MSC group compared to the CB group. Notably, in this study, the expression of the Col III protein was significantly decreased in the MSC group compared to the CB group, which may be due to the small proportion of Col III in the ECM. Matrix metalloproteinases (MMP-1 and MMP-9) are zinc-dependent endopeptidases that degrade ECM [42]. Mitochondrial transfer significantly reduced the expression of MMP-1 and MMP-9; however, this statistical significance was weakened after CB intervention.

According to the instructions of the reagent provider, the potential induction time of BMSC trilineage differentiation was approximately 3–4 weeks. Based on a previous report [35], we used days 5 and 14 to reduce the impact of differentiation. This study assessed tendon healing using H&E, PSR, and Masson staining. Compared with the PBS group, the MSC group exhibited a higher cell density, a regular fiber arrangement, and dense fiber bundles. The pathological outcome of the tendons in the CB treatment group was worse than that in the MSC group. This finding confirms that mitochondrial transfer from MSCs to collagenase-induced Achilles tendon tissues can significantly improve the progress of Achilles tendinopathy.

Excessive loading and overuse of tendons are among the major causes of tendinopathy and result in overexpression of ROS [48]. Mitochondrial dysfunction and ROS generation trigger a series of detrimental consequences for the cell [49]. The DCFH probe is insensitive to intracellular ROS and is affected by enzymatic oxidation and redox-active metals; hence, we further measured the level of mitochondrial ROS using the MitoSOX probe. Oxidative stress, depolarization of ΔΨm, and opening of mPTP caused by H_2_O_2_ treatment induce activation of the mitochondria-mediated tenocyte apoptotic pathway, contributing to the progression of tendinopathy [29]. Numerous studies have demonstrated that MSCs transfer healthy mitochondria to damaged acceptor cells and improve mitochondrial function and cellular performance, including OCR, ROS, ΔΨm, ATP, cell proliferation, and apoptosis, such as in stroke-like episodes fibroblasts [31], airway smooth muscle cells [18], and chondrocytes [50]. Our findings presented similar results. Furthermore, we also found that H_2_O_2_ blunted mitochondrial respiration and triggered apoptosis in co-cultured tenocytes. Interestingly, when mitochondria were transferred from MSCs to injured tenocytes, the negative effects of in vitro H_2_O_2_ induction were partially reversed. Tenocyte proliferation and mitochondrion-dependent viability were significantly improved due to improved mitochondrial bioenergetics.

An imbalance between mitochondrial fusion and fission occurs when mitochondria do not function properly, causing excessive ROS generation and leading to activation of the apoptosis pathway [23]. Injuries can trigger mitochondrial fission, and the mitochondrial fission and fusion pathways are interconnected [51]. Transferring MSC mitochondria to damaged tenocytes resulted in a decrease in fission (Drp1) and an increase in fusion (Mfn2) levels.

As inflammation is an underlying mechanism of tendinopathy pathogenesis [23], mitochondrial transfer suppressed the levels of pro-inflammatory markers (IL-1β, IL-6, and TNF-α), revealing a promising therapeutic strategy against inflammatory. Moreover, the presence of fewer macrophages results in a lesser release of pro-inflammatory cytokines and less secondary damage [52]. We also found that the infiltration of macrophages (CD68) into the AT tendon was downregulated by mitochondrial transfer.

ROS can directly trigger the opening of mPTP and decrease in ΔΨm, leading to mitochondrial caspase-dependent apoptosis pathway activation [53]. Excess ROS levels cause cardiolipin peroxidation. Oxidized cardiolipin on the outer mitochondrial membrane can also recruit Bax to trigger the mitochondrial permeability transition, which releases Cyt-c from the mitochondria to initiate the activation of apoptosome, the activation of caspase 3, and the apoptosis [54]. The opening of mPTP has been linked to Cyt-c release and is important in initiating the mitochondrial apoptotic pathway [55]. According to the above theories, mitochondrial transfer significantly attenuated H_2_O_2_-induced increases in caspase 3, caspase 9, Cyt-c, and Bax levels in co-cultured tenocytes. Increased mitochondrial permeability leads to cytosolic release of pro-apoptotic factors (Cyt-c, Smac/DIABLO, and AIF) from mitochondria [56]. Cyt-c binds to apoptosis-inducing protease-1 (Apaf-1) and induces caspase-dependent apoptosis [57]. Mitochondrial AIF is released into the cytosol and translocated to the nucleus, where it triggers condensation of chromatin and DNA fragmentation [58]. Smac/DIABLO directly bind to the inhibitor of apoptosis proteins (IAPs) and promote apoptosis [59]. This study revealed that the release of mitochondrial function proteins (Cyt-c, AIF, and Smac) further promoted apoptosis, and mitochondrial transfer can reduce the release of mitochondrial pro-apoptotic proteins and reduce apoptosis.

Wang et al. [50] revealed that mitochondrial transfer from BMSCs to osteoarthritic chondrocytes increases ΔΨm, ATP, and cell survival. Furthermore, mRNA expression of the proliferation marker Ki67 increased and cell survival was promoted in injured tenocytes following the administration of mitochondria from MSCs. Consistent with a previous study [60], our results further confirmed that blocking MSC-derived mitochondrial transfer by cytochalasin B eliminated the beneficial effect of MSCs on the survival of damaged tenocytes. Therefore, we suggest that mitochondrial transfer is effective in preventing tenocytes from undergoing mitochondrion-driven apoptosis.

One cause of AT is repeated long-term tendon tensile mechanical injury, resulting in an increased demand for intracellular energy. Once mitochondrial function is abnormal, it will contribute to the development of tendinopathy, and recovery and activation of mitochondrial function will help restore tissue revitalization and rejuvenation [61]. Chris et al. [6] reported that tendon progenitor cells (TPCs) have seven subpopulations, which have high mitochondrial expression genes in normal tendon tissues and low expression in tendon tissues of tendinopathy. Using TPCs provides a potential pathway for tendon regeneration. Whether TPCs can obtain more healthy exogenous mitochondria through the mitochondrial transfer mechanism, restore cell function, and contribute to the treatment of tendinopathy requires more in-depth research.

This study had limitations. First, although we have confirmed that mitochondrial transfer can improve tenocyte apoptosis, mitochondrial transfer promotion strategies have not been further studied. Emerging evidence has demonstrated that overexpression of mitochondrial motor protein Rho-GTPase 1 (Miro1) can enhance mitochondrial transfer [11, 17, 44], but the promotion of mitochondrial transfer from MSCs to tenocytes by Miro1 requires further evaluation. Second, increasing evidence has confirmed that TNTs are channels of mitochondrial transfer [62–64], but our study has not verified this. Future research should focus on promoting TNTs formation between donor and recipient cells. Third, we only used CB to block mitochondrial transfer. However, the differences in some indicators (e.g. relative apoptosis genes and proteins) between the CB group and the H_2_O_2_- or collagenase-induced groups were also statistically significant. Other ways of connecting MSCs and tenocytes or tissues, such as gap junctions and cell fusion, require further study [31]. Forth, although there has been a fair amount of preclinical research on stem cells and mitochondrial transfer as treatments for AT, further studies in primate models are still needed before clinical application of MSC to patients. Finally, biomechanical evaluations of the Achilles tendon and behavioral outcomes, including pain response and gait, were not performed.

## Conclusions

This study provides a novel mechanism for the therapeutic effects of BMSCs in Achilles tendinopathy. Mitochondrial transfer from BMSCs to damaged tenocytes in vitro and in vivo restores tenocyte mitochondrial function, reduces cell apoptosis, modulates inflammation, and promotes tendon healing. Collectively, our results highlight the therapeutic effects of mitochondrial transfer from MSCs on rat tendinopathy.

## Supplementary Information

**Additional file 1: Fig. S1.** Characterization of BMSCs. A Flow cytometry analysis revealed that 98.21% of cells expressed CD90 and 95.52% expressed CD44. Meanwhile, only 3.87% and 0.71% expressed CD34 and CD45, respectively. B To further characterize MSCs, cells were induced to differentiate into adipogenic, osteogenic, and chondrogenic lineages. Representative microscopic images of differentiation assays for osteogenic, chondrogenic, and adipogenicpathways. Scale bars: 200 × , 20 µm; 40 × , 200 µm; 400 × , 10 µm.

**Additional file 2: Fig. S2.** Characterization of tenocytes. A Cell IF staining was performed to detect Col I and Col III. Scale bars: 100 × , 200 μm; 400 × , 100 μm. B RT-qPCR analysis revealed that, compared to adipose tissue, tendon-specific markers mRNAwere highly expressed in primary cultured tenocytes of the Achilles tendon. Data represent mean ± SD. Statistical significance of the differences between two groups was determined by Student's t-test. ****P < 0.0001.

**Additional file 3: Fig. S3.** The H_2_O_2_ concentrationwas optimized for the assay conditions by the CCK-8 experiment. A Six H_2_O_2_ concentrations were selected. B Nine H_2_O_2_ concentrations were selected. CCK-8, Cell Counting Kit-8. Data represent mean ± SD. Statistical significance of the differences between H_2_O_2_ and H_2_O_2_ was determined by Student's t-test. ***P < 0.001.

**Additional file 4. Table S1.** PCR primer information used in this study.

**Additional file 5. Table S2.** Antibodies used in specific applications.

**Additional file 6. Fig. S4.** Mitochondria successfully transferred from MSCs to H2O2-induced tenocytes.

**Additional file 7.** The original gel/blot images are displayed.

## Data Availability

All data generated and analyzed during this study are available from the corresponding author on reasonable request.

## Abbreviations

AIF: Apoptosis-inducing factor
AT: Achilles tendinopathy
ATP: Adenosine triphosphate
Bax: BCL2-associated X
Bcl-2: B-cell lymphoma 2
BMSCs: Bone marrow mesenchymal stem cells
CB: Cytochalasin B
CCK-8: Cell Counting Kit-8
Col I: Collagen type I
Col III: Collagen type III
CTV: CellTracker™ Violet
Cyt-c: Cytochrome c
Drp1: Dynamin-related protein 1
H_2_O_2_: Hydrogen peroxide
IF: Immunofluorescence
IHC: Immunohistochemistry
IL-1β: Interleukin-1β
IL-6: Interleukin-6
intra-ROS: Intracellular ROS
Mfn2: Mitofusin 2
mito-ROS: Mitochondrial ROS
MMP-1: Matrix metalloproteinase 1
MMP-9: Matrix metalloproteinase 9
mPTP: Mitochondrial permeability transition pore
MSCs: Mesenchymal stem cells
OCR: Oxygen consumption rate
PBS: Phosphate-buffered saline
PSR: Picrosirius red
RFI: Relative fluorescence intensity
ROS: Reactive oxygen species
Rot: Rotenone
Scx: Scleraxis
TN-C: Tenascin C
Tnmd: Tenomodulin
TNTs: Tunneling nanotubes
TNF-α: Tumor necrosis factor-α
ΔΨm: Mitochondrial membrane potential
