# Supplementary figures and images for: Mitochondrial transfer from bone mesenchymal stem cells protects against tendinopathy both in vitro and in vivo
Source: Stem Cell Res Ther. 2023 Apr 26;14:104. doi: 10.1186/s13287-023-03329-0 (PMC10134653; doi:10.1186/s13287-023-03329-0)

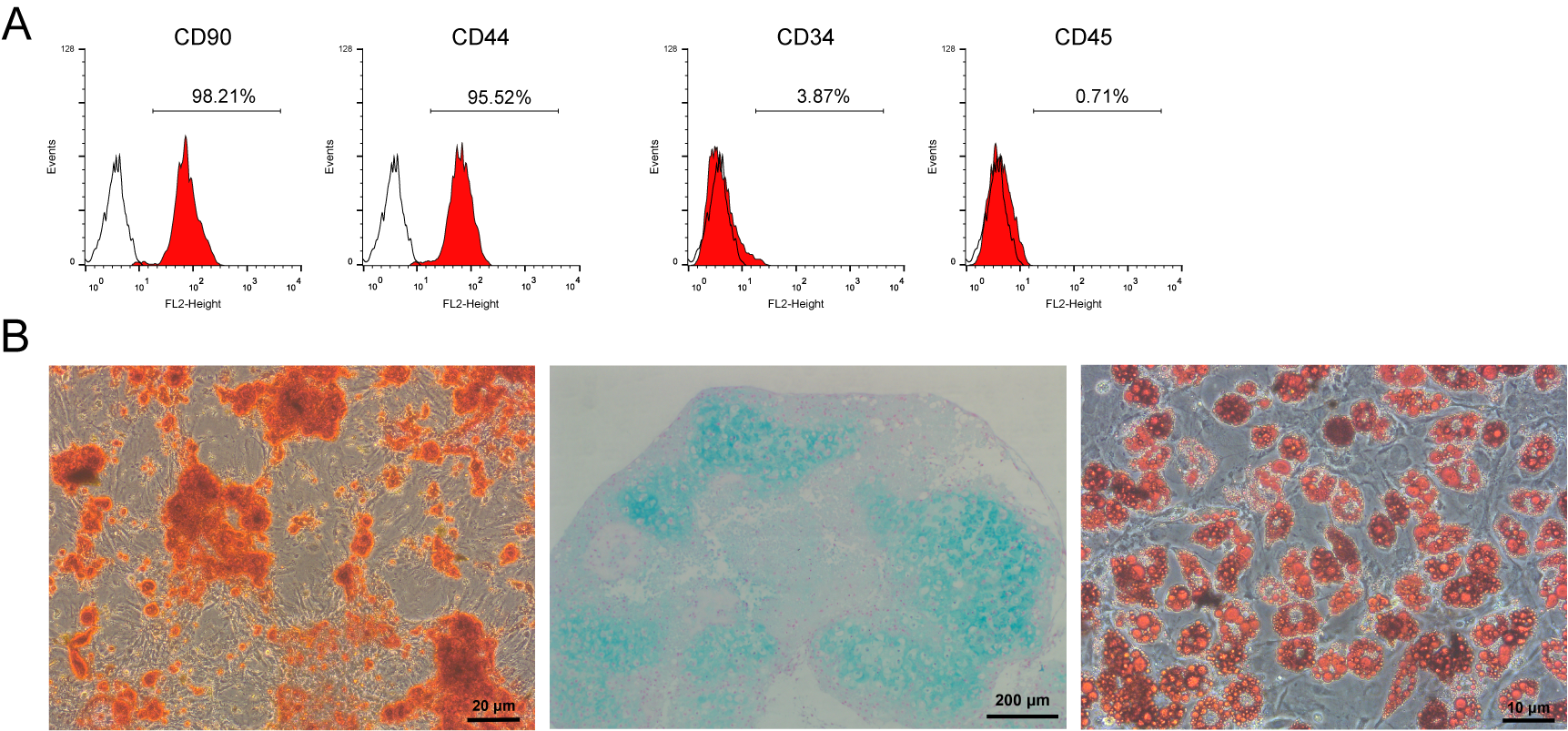

Supplement: Supplementary file 1 — Additional file 1: Fig. S1. Characterization of BMSCs. A Flow cytometry analysis revealed that 98.21% of cells expressed CD90 and 95.52% expressed CD44. Meanwhile, only 3.87% and 0.71% expressed CD34 and CD45, respectively. B To further characterize MSCs, cells were induced to differentiate into adipogenic, osteogenic, and chondrogenic lineages. Representative microscopic images of differentiation assays for osteogenic, chondrogenic, and adipogenicpathways. Scale bars: 200 × , 20 µm; 40 × , 200 µm; 400 × , 10 µm. [file 13287_2023_3329_MOESM1_ESM.tif]

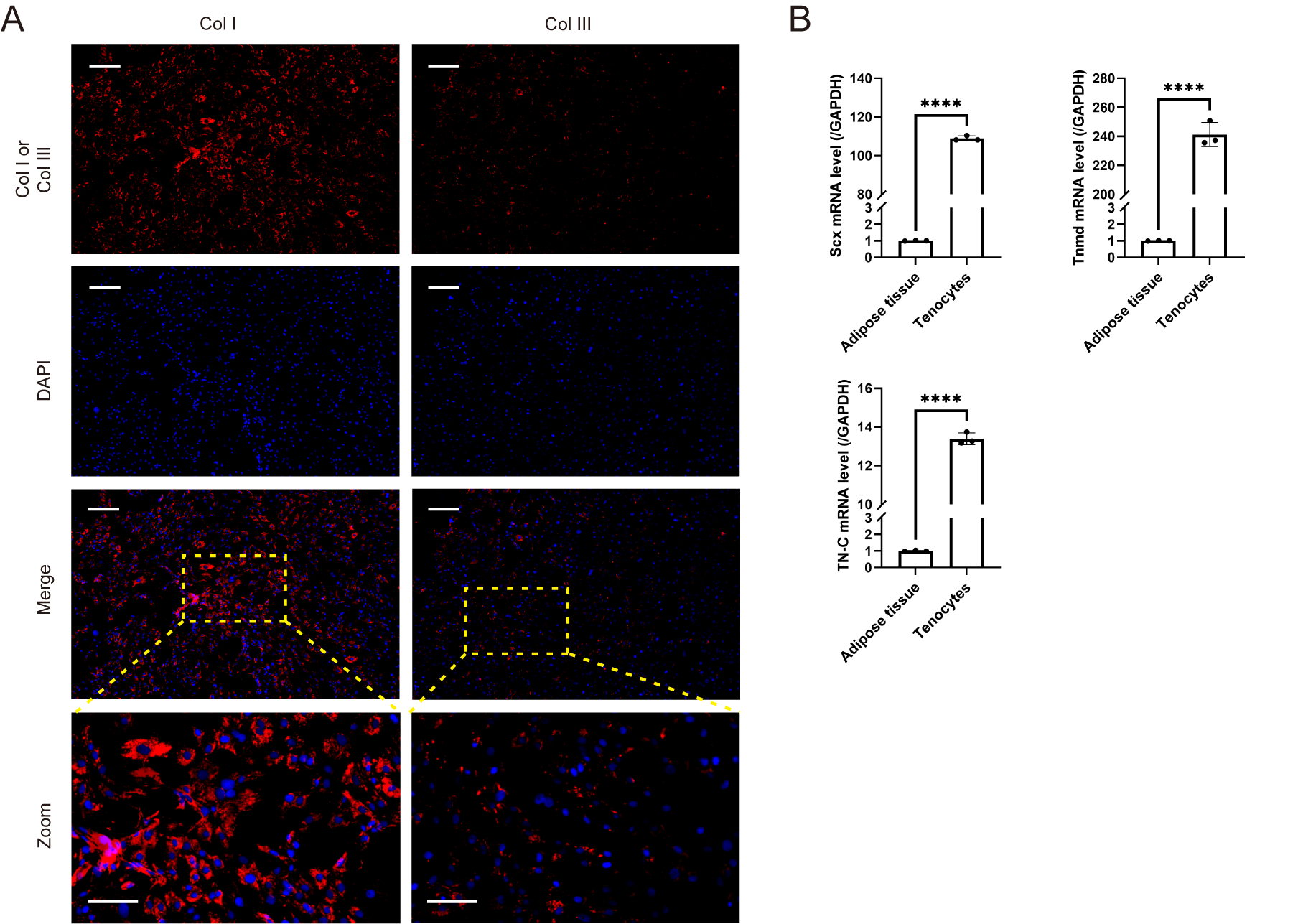

Supplement: Supplementary file 2 — Additional file 2: Fig. S2. Characterization of tenocytes. A Cell IF staining was performed to detect Col I and Col III. Scale bars: 100 × , 200 μm; 400 × , 100 μm. B RT-qPCR analysis revealed that, compared to adipose tissue, tendon-specific markers mRNAwere highly expressed in primary cultured tenocytes of the Achilles tendon. Data represent mean ± SD. Statistical significance of the differences between two groups was determined by Student's t-test. ****P < 0.0001. [file 13287_2023_3329_MOESM2_ESM.tif]

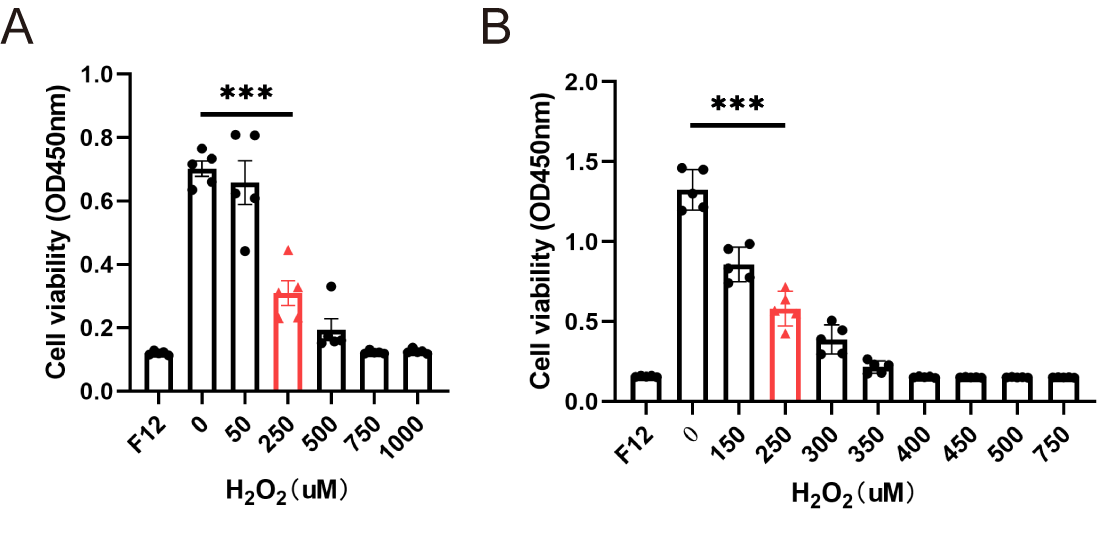

Supplement: Supplementary file 3 — Additional file 3: Fig. S3. The H2O2 concentrationwas optimized for the assay conditions by the CCK-8 experiment. A Six H2O2 concentrations were selected. B Nine H2O2 concentrations were selected. CCK-8, Cell Counting Kit-8. Data represent mean ± SD. Statistical significance of the differences between H2O2 and H2O2 was determined by Student's t-test. ***P < 0.001. [file 13287_2023_3329_MOESM3_ESM.tif]
