# Supplementary material for: Mitochondrial transfer from bone mesenchymal stem cells protects against tendinopathy both in vitro and in vivo
Source: Stem Cell Res Ther. 2023 Apr 26;14:104. doi: 10.1186/s13287-023-03329-0 (PMC10134653; doi:10.1186/s13287-023-03329-0)
Supplement: Supplementary file 4 — Additional file 4. Table S1. PCR primer information used in this study. [file 13287_2023_3329_MOESM4_ESM.docx]

**Additional file 4:** **Table S1.** PCR primer information used in this study.

| Gene | Primer | Sequence (5’-3’) |
| --- | --- | --- |
| Scx | Forward | TCTGCACCTTCTGCCTCA |
|  | Reverse | CACCTCTTGGCTGCTGTG |
| TN-C | Forward | AGCAAACTGATCCAAACCA |
|  | Reverse | TGCGTCTCAGGAAAACG |
| Tnmd | Forward | AGGACTTTGAGGAGGATGG |
|  | Reverse | CTCGCTTGCTTGTCTGG |
| TNF-α | Forward | GGTCCCAACAAGGAGGAGAAGT |
|  | Reverse | GGTTTGCTACGACGTGGGCTA |
| Ki-67 | Forward | CCATTAACAAGAGTGAGGGAGTG |
|  | Reverse | TGAGTGGAGTATTAGGAGGCAAG |
| Caspase 3 | Forward | CTGGACTGCGGTATTGAGACA |
|  | Reverse | CGGGTGCGGTAGAGTAAGC |
| Caspase 9 | Forward | CCACTGCCTCATCATCAACAAC |
|  | Reverse | GCCGTGACCATTTTCTTAGCAG |
| Col1α1 | Forward | CGTGGAAACCTGATGTATGCTTG |
|  | Reverse | CCTATGACTTCTGCGTCTGGTGA |
| Col3α1 | Forward | CGAGGTAACAGAGGTGAAAGAGG |
|  | Reverse | TTTCACCTCCAACTCCAGCAAT |
| Bcl-2 | Forward | TTGTGGCCTTCTTTGAGTTCG |
|  | Reverse | GCATCCCAGCCTCCGTTAT |
| Bax | Forward | GGGTGGTTGCCCTTTTCTACTT |
|  | Reverse | GAAGTCCAGTGTCCAGCCCAT |
| Cyt-c | Forward | CTTGGGCTAGAGAGCGGGA |
|  | Reverse | GGTATCCTCTCCCCAGGTGAT |
| GAPDH | Forward | CAAGAAGGTGGTGAAGCAG |
|  | Reverse | CAAAGGTGGAAGAATGGG |

Abbreviations: Scx, Scleraxis; TN-C, Tenascin-C; Tnmd, Tenomodulin; TNF-α, tumor necrosis factor-α; Col1a1, collagen type 1α1; Col3α1, collagen type 3α1; Cyt-c, Cytochrome C.
