# Supplementary material for: Mitochondrial transfer from bone mesenchymal stem cells protects against tendinopathy both in vitro and in vivo
Source: Stem Cell Res Ther. 2023 Apr 26;14:104. doi: 10.1186/s13287-023-03329-0 (PMC10134653; doi:10.1186/s13287-023-03329-0)
Supplement: Supplementary file 5 — Additional file 5. Table S2. Antibodies used in specific applications. [file 13287_2023_3329_MOESM5_ESM.docx]

**Additional file 5: Table S2.** Antibodies used in specific applications.

| **Primary Antibody**  **(Manufacture, catalog #, dilution used)** | **Second Antibody**  **(Manufacture, catalog #, dilution used)** | **Concentrations of SDS-PAGE gel** | **Application** |
| --- | --- | --- | --- |
| **Western blot** | | | |
| rabbit polyclonal anti-Bcl-2 antibody  (Abcam, ab196495, 1:500) | HRP conjugated Goat Anti-Rabbit IgG (H+L) (Servicebio, GB23303, 1:2000) | 12% | Fig. 1K |
| rabbit polyclonal anti-Bax antibody  (Servicebio, GB11690, 1:500) | HRP conjugated Goat Anti-Rabbit IgG (H+L) (Servicebio, GB23303, 1:2000) | 12% | Fig. 1K |
| rabbit polyclonal anti-caspase 3 antibody  (Servicebio, GB11767C, 1:1000) | HRP conjugated Goat Anti-Rabbit IgG (H+L) (Servicebio, GB23303, 1:3000) | 10% | Fig. 1L |
| rabbit polyclonal anti-caspase 9 antibody  (Servicebio, GB11053-1, 1:800) | HRP conjugated Goat Anti-Rabbit IgG (H+L) (Servicebio, GB23303, 1:2000) | 10% | Fig. 1L |
| rabbit polyclonal anti-cytochrome C antibody  (Servicebio, GB11080, 1:800) | HRP conjugated Goat Anti-Rabbit IgG (H+L) (Servicebio, GB23303, 1:2000) | 15% | Fig. 1J |
| rabbit monoclonal anti-AIF antibody  (Beyotime, AF1273, 1:1000) | HRP conjugated Goat Anti-Rabbit IgG (H+L) (Beyotime, A0208, 1:2000) | 8% | Fig. 1J |
| rabbit monoclonal anti-Smac/DIABLO antibody  (Beyotime, AF2092, 1:1000) | HRP conjugated Goat Anti-Rabbit IgG (H+L) (Beyotime, A0208, 1:2000) | 12% | Fig. 1J |
| rabbit monoclonal anti-Mfn2 antibody  (Abcam, ab124773, 1:5000) | HRP conjugated Goat Anti-Rabbit IgG (H+L) (Servicebio, GB23303, 1:2000) | 8% | Fig. 2F |
| rabbit monoclonal anti-Drp1 antibody  (CST, 8570S, 1:1000) | HRP conjugated Goat Anti-Rabbit IgG (H+L) (Servicebio, GB23303, 1:2000) | 8% | Fig. 2F |
| rabbit polyclonal anti-tenomodulin antibody  (Abcam, ab203676, 1:1000) | HRP conjugated Goat Anti-Rabbit IgG (H+L) (Servicebio, GB23303, 1:3000) | 10% | Fig. 3D |
| rabbit polyclonal anti-MMP-1 antibody  (Affinity, DF6325, 1:1000) | HRP conjugated Goat Anti-Rabbit IgG (H+L) (Servicebio, GB23303, 1:2000) | 10% | Fig. 3E |
| rabbit polyclonal anti-collagen I antibody  (Servicebio, GB11022, 1:1000) | HRP conjugated Goat Anti-Rabbit IgG (H+L) (Servicebio, GB23303, 1:3000) | 6% | Fig. 3F |
| rabbit polyclonal anti-collagen III antibody  (Servicebio, GB11023, 1:500) | HRP conjugated Goat Anti-Rabbit IgG (H+L) (Servicebio, GB23303, 1:2000) | 6% | Fig. 3F |
| rabbit polyclonal anti-β-Actin antibody  (Servicebio, GB15003, 1:2000) | HRP conjugated Goat Anti-Rabbit IgG (H+L) (Servicebio, GB23303, 1:3000) | 10% | Fig. 1J-L, Fig. 2F, Fig. 3D-F |
| **Immunofluorescent** | | | |
| rabbit polyclonal anti-collagen I antibody  (Servicebio, GB11022, 1:1000) | FITC conjugated Goat Anti-Rabbit IgG (H+L) (Servicebio, GB22303, 1:100) | N/A | Sup Fig. 2A,  Fig. 6F |
| rabbit polyclonal anti-collagen III antibody  (Servicebio, GB11023, 1:500) | FITC conjugated Goat Anti-Rabbit IgG (H+L) (Servicebio, GB22303, 1:50) | N/A | Sup Fig. 2A,  Fig. 6G |
| **Immunohistochemistry** | | | |
| rabbit polyclonal anti-CD68 antibody  (Servicebio, GB113109, 1:200) | HRP conjugated Goat Anti-Rabbit IgG (H+L) (Servicebio, GB23303, 1:500) | N/A | Fig. 6D |
| rabbit polyclonal anti-MMP-9 antibody  (Servicebio, GB11132, 1:1000) | HRP conjugated Goat Anti-Rabbit IgG (H+L) (Servicebio, GB23303, 1:400) | N/A | Fig. 6E |

Abbreviations: AIF: apoptosis-inducing factor; Bcl-2, B-cell lymphoma 2; Bax, BCL2-Associated X; Mfn2, Mitofusin 2; Drp1, Dynamin-related protein 1; MMP-1, Matrix metalloproteinase-1; MMP-9, Matrix metalloproteinase-9; HRP, horseradish peroxidase; N/A, not applicable.
