# Supplementary material for: Mitochondrial transfer from bone mesenchymal stem cells protects against tendinopathy both in vitro and in vivo
Source: Stem Cell Res Ther. 2023 Apr 26;14:104. doi: 10.1186/s13287-023-03329-0 (PMC10134653; doi:10.1186/s13287-023-03329-0)
Supplement: Supplementary file 6 — Additional file 6. Fig. S4. Mitochondria successfully transferred from MSCs to H2O2-induced tenocytes. [file 13287_2023_3329_MOESM6_ESM.docx]

**
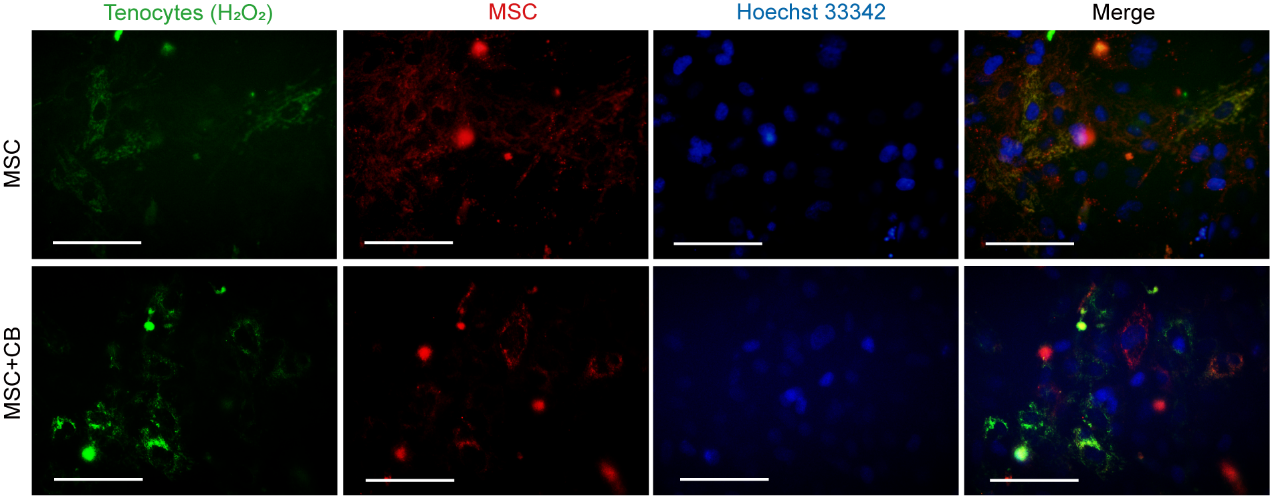
**

**Additional file 6: Fig. S4** Mitochondria successfully transferred from MSC to H_2_O_2_-induced tenocytes. Representative fluorescence images showing MitoTracker Red CMXRos-labeled MSC mitochondria (red) in tenocytes (green) *in vitro*. Nuclei are counterstained with Hoechst 33342 (blue). Scale bar: 100×, 100 µm.
