# Supplementary material for: Mitochondrial transfer from bone mesenchymal stem cells protects against tendinopathy both in vitro and in vivo
Source: Stem Cell Res Ther. 2023 Apr 26;14:104. doi: 10.1186/s13287-023-03329-0 (PMC10134653; doi:10.1186/s13287-023-03329-0)

**Additional file 7**: The original gel/blot images are displayed below.

Fig.1

AIF (67 kDa)


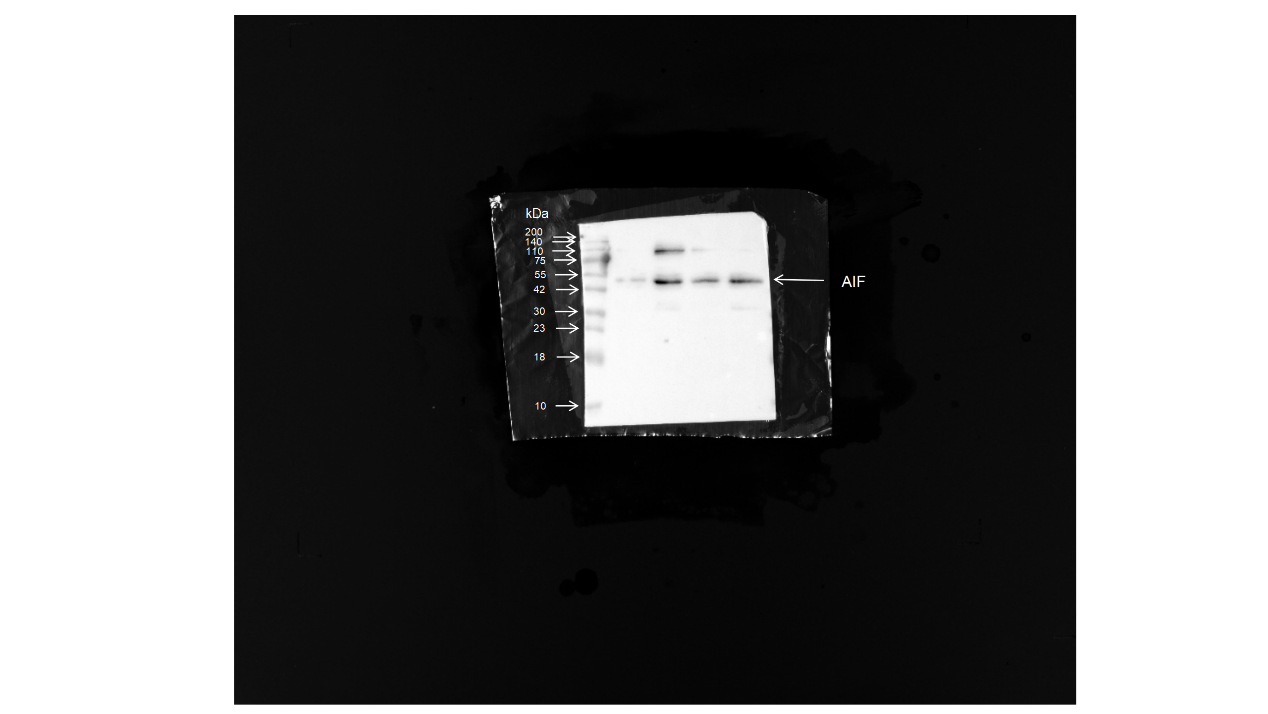


Smac (21 kDa)


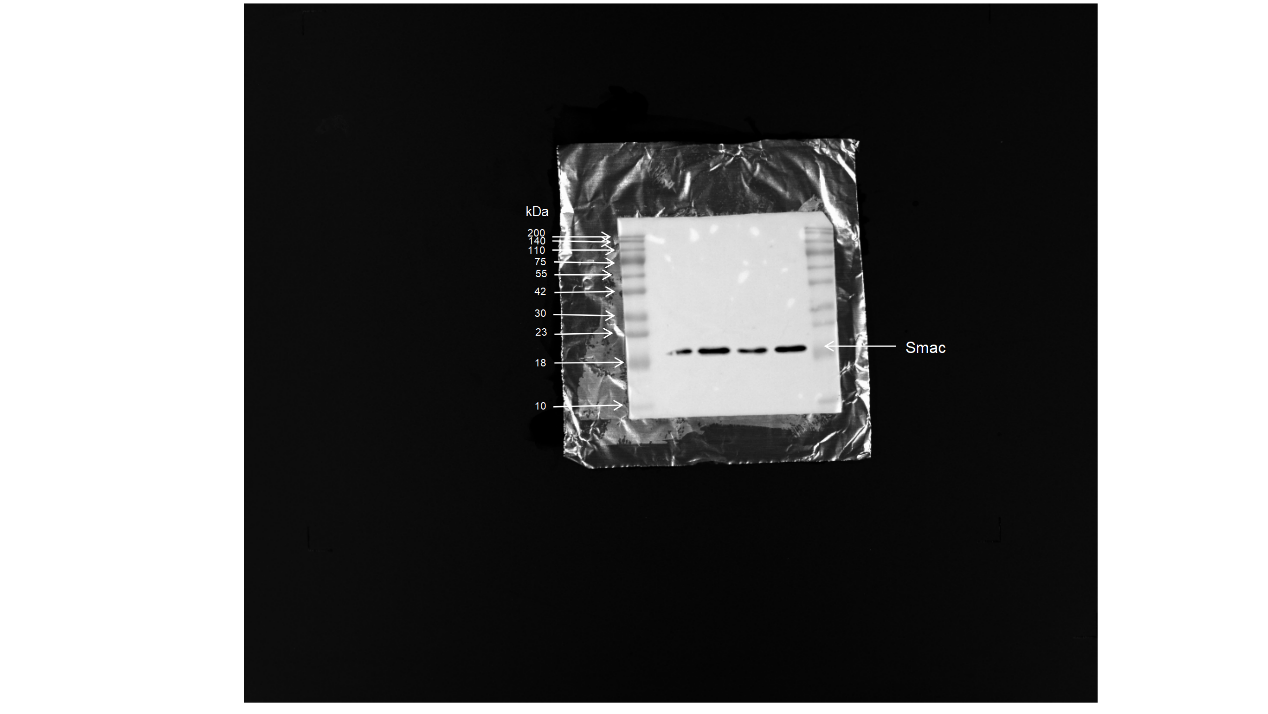


β-Actin (45 kDa)


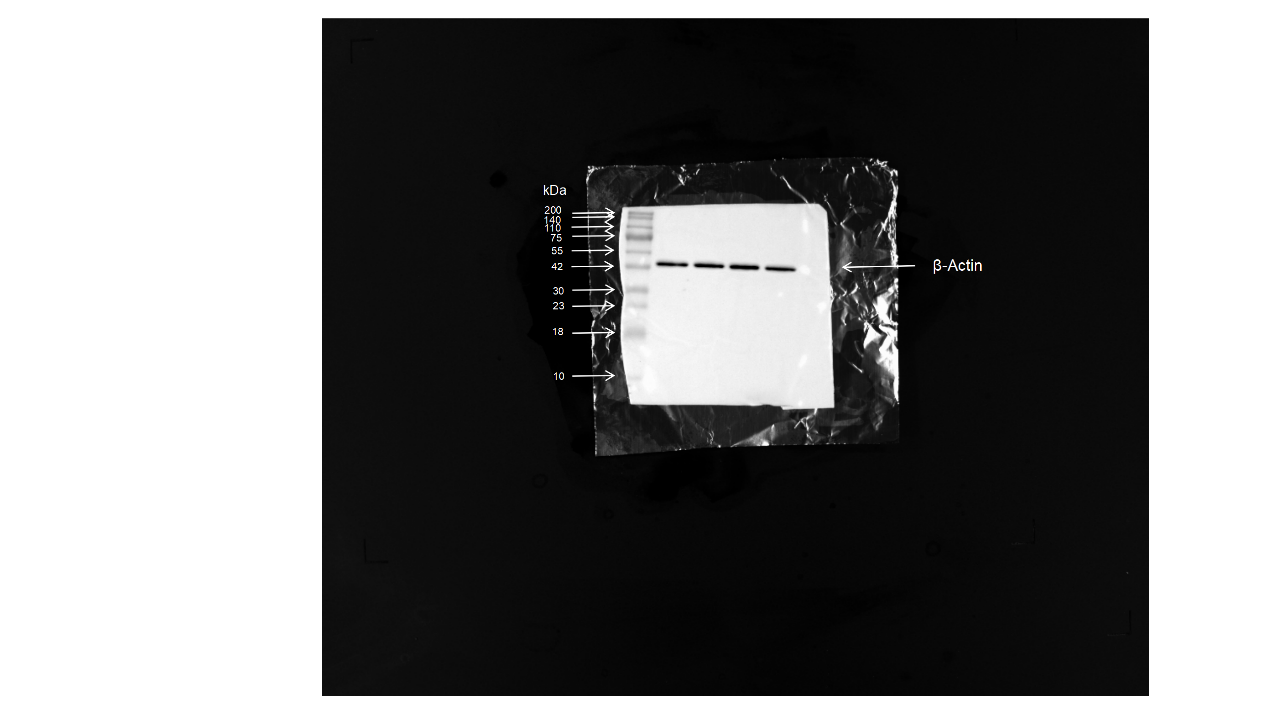


Cyt-c (21 kDa)


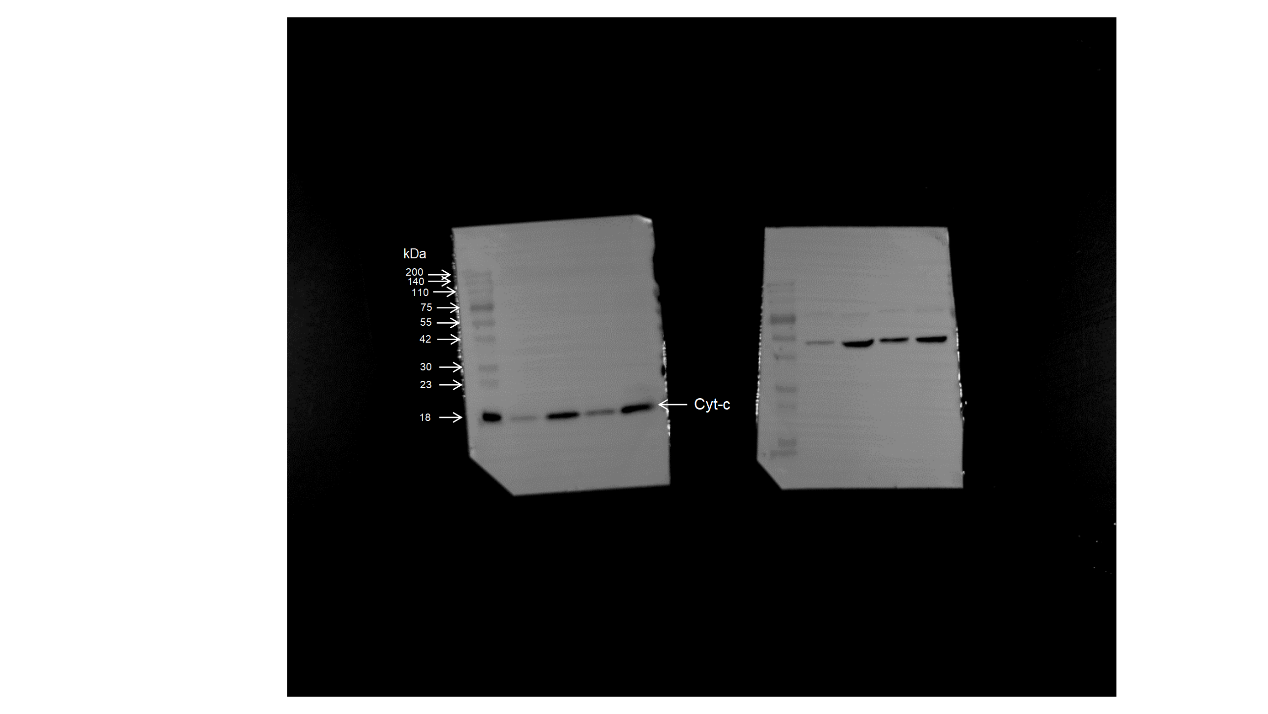


β-Actin (45 kDa)


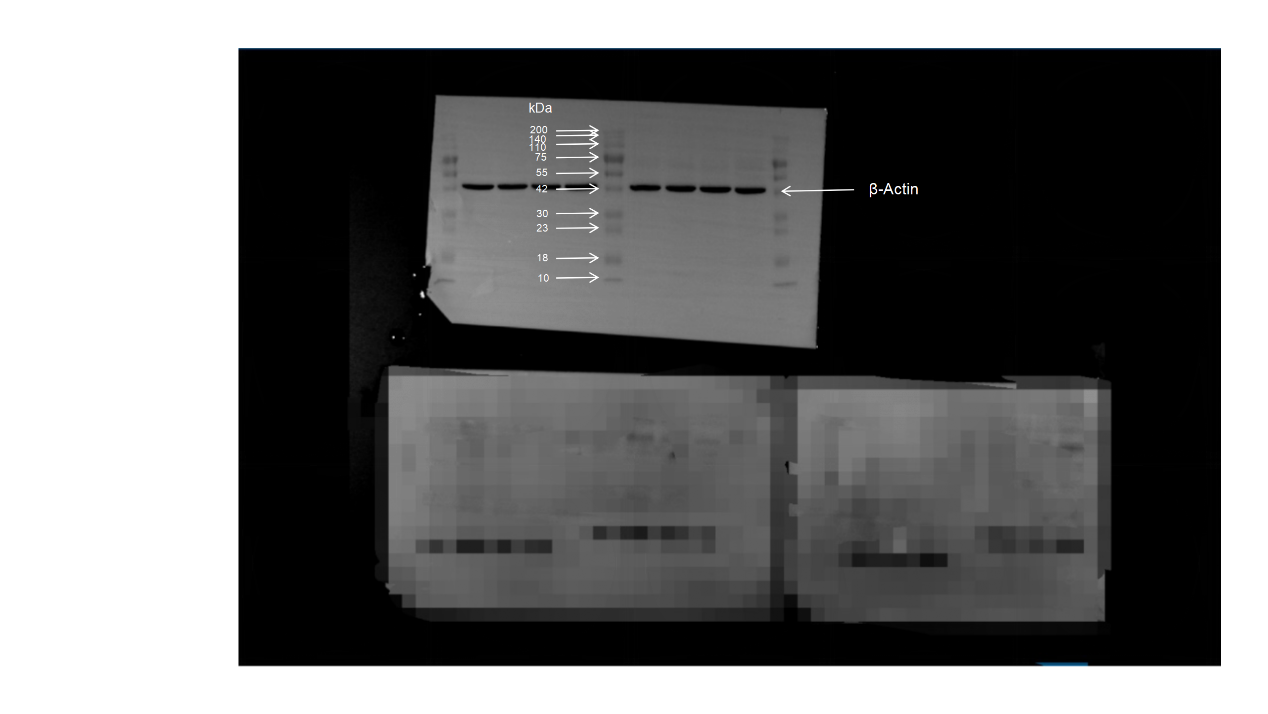


Bcl-2 (26 kDa)


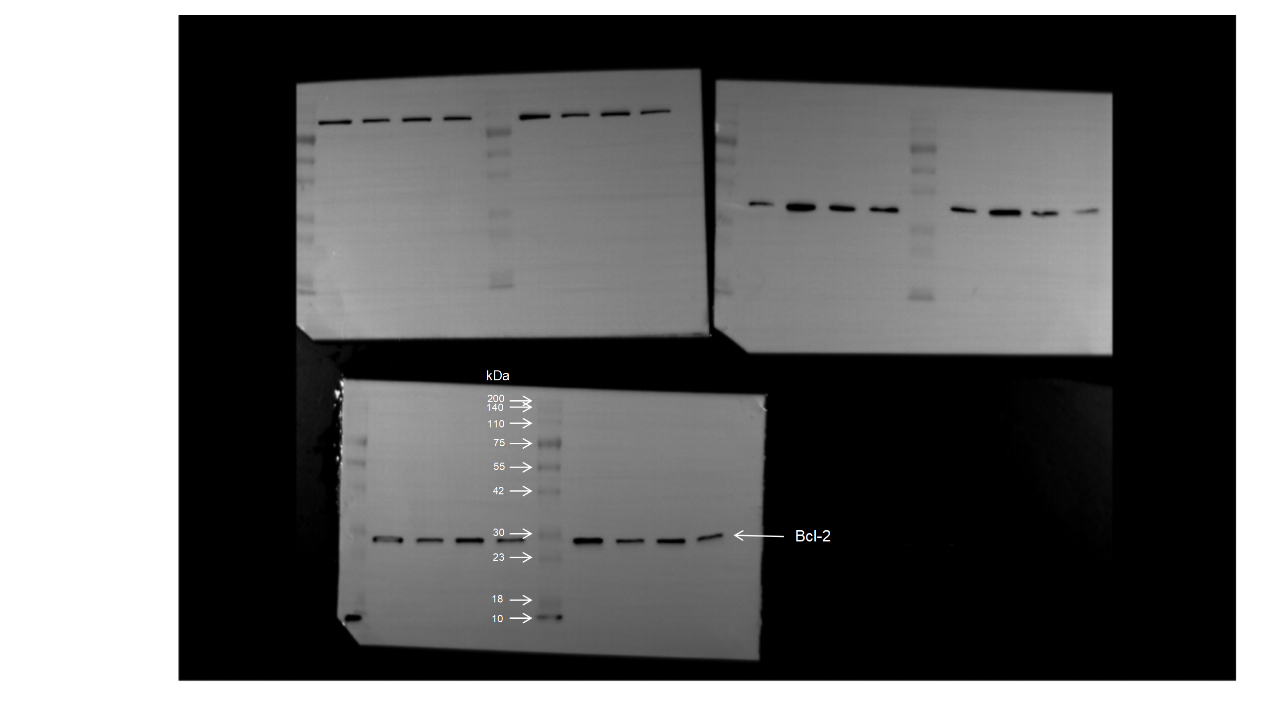


Bax (21 kDa)


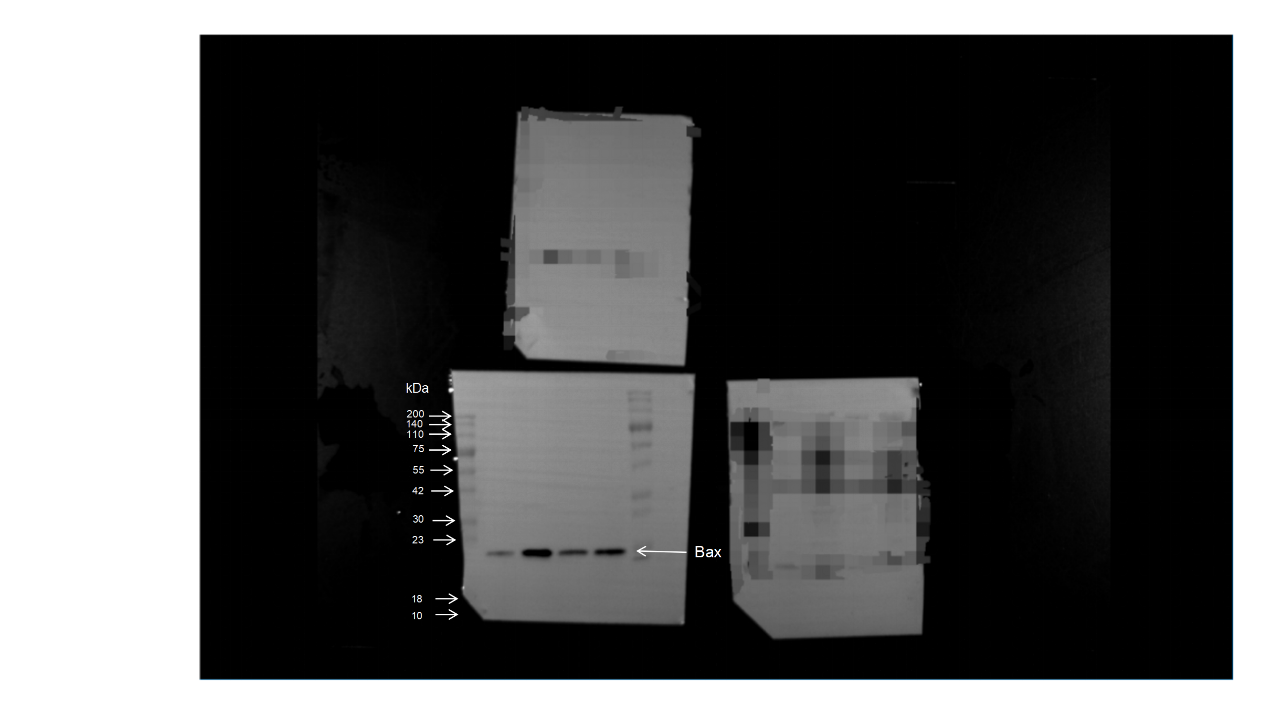


β-Actin (45 kDa)


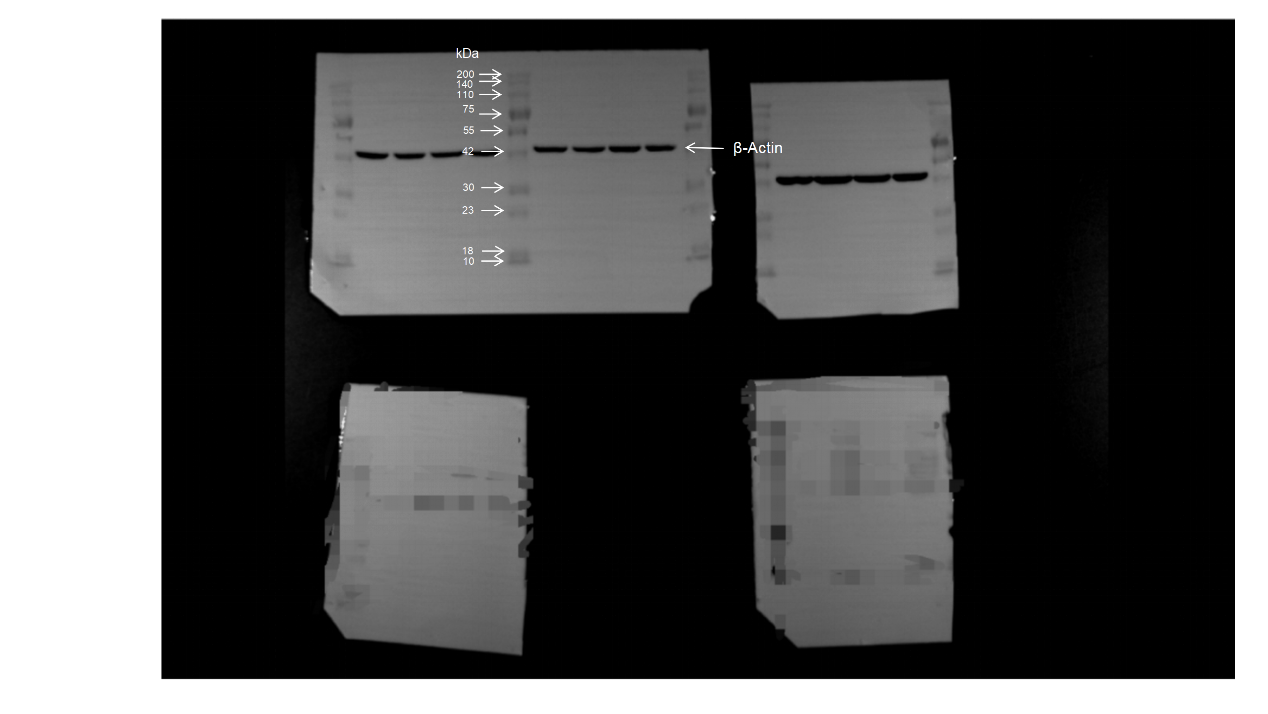


Caspase 3 (35 kDa)


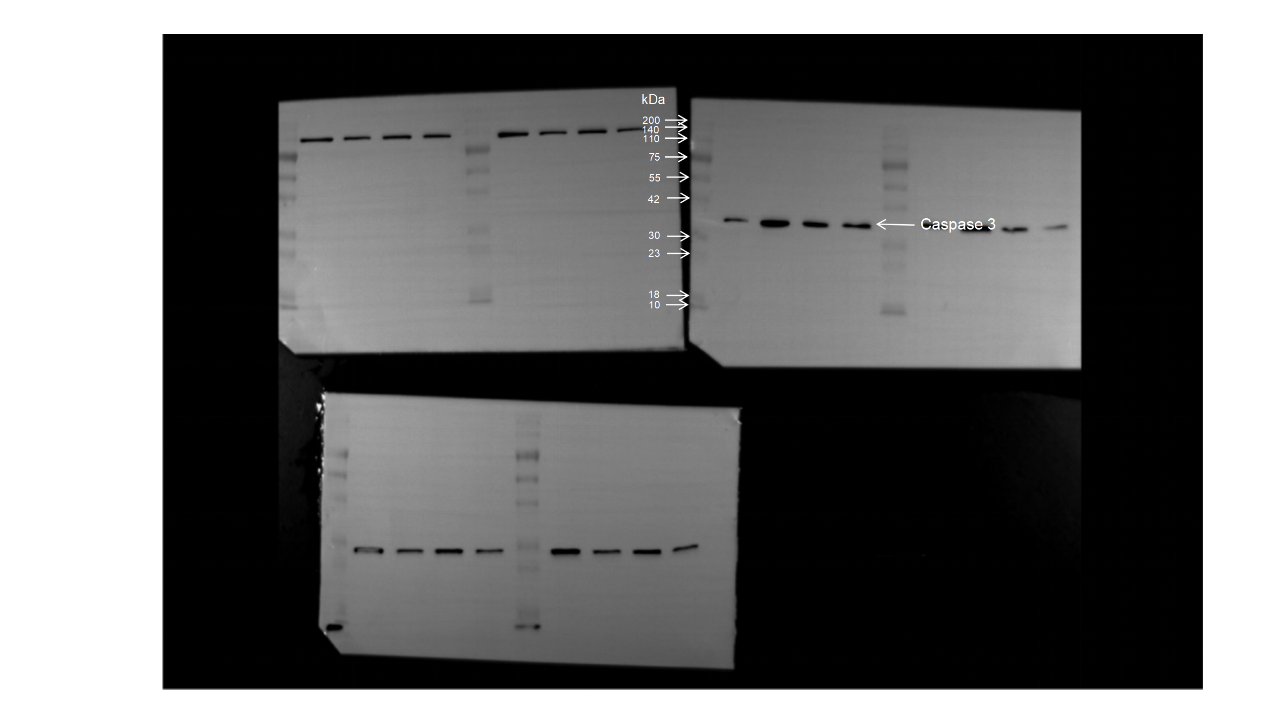


Caspase 9 (35/46 kDa)


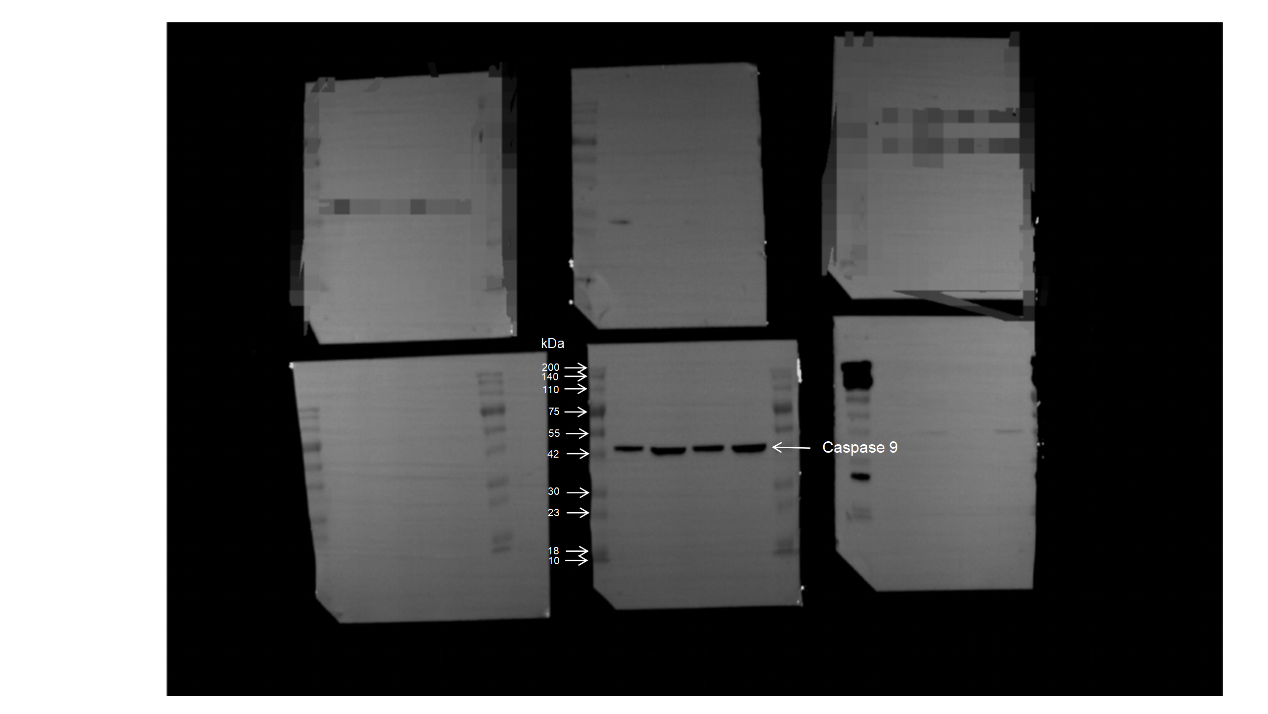


β-Actin (45 kDa)


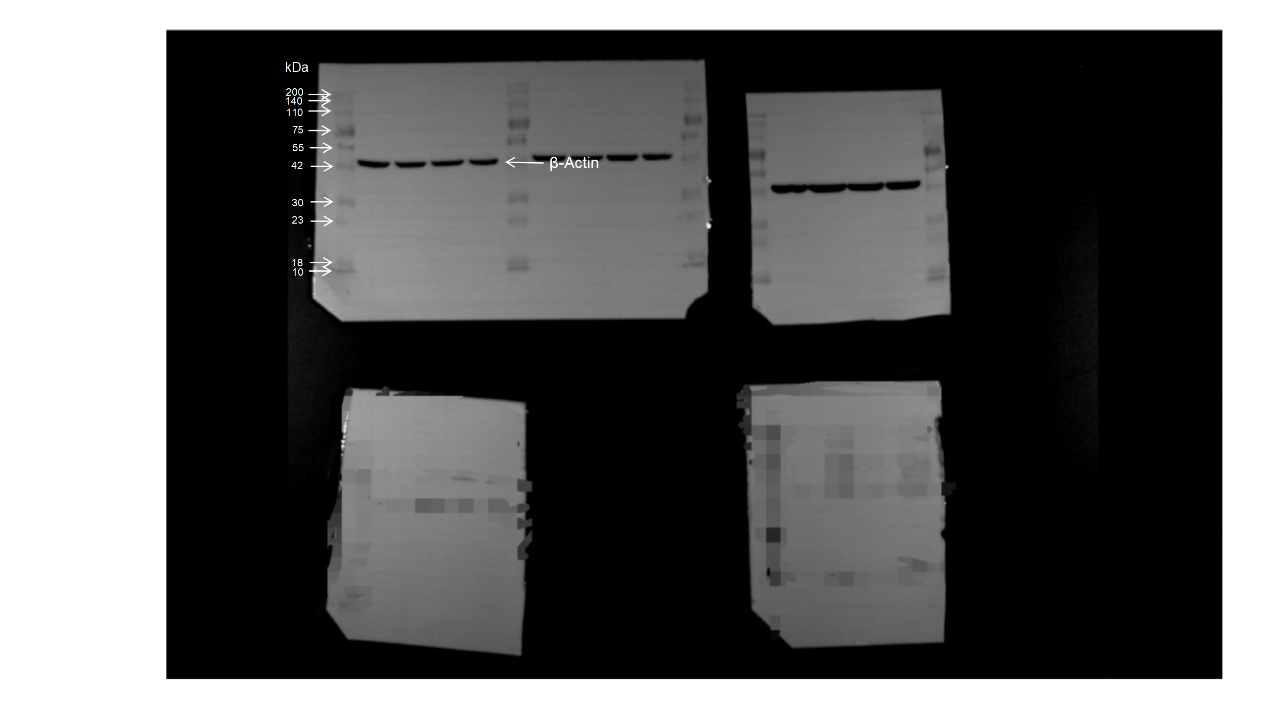


Fig.2

Drp1 (80 kDa)


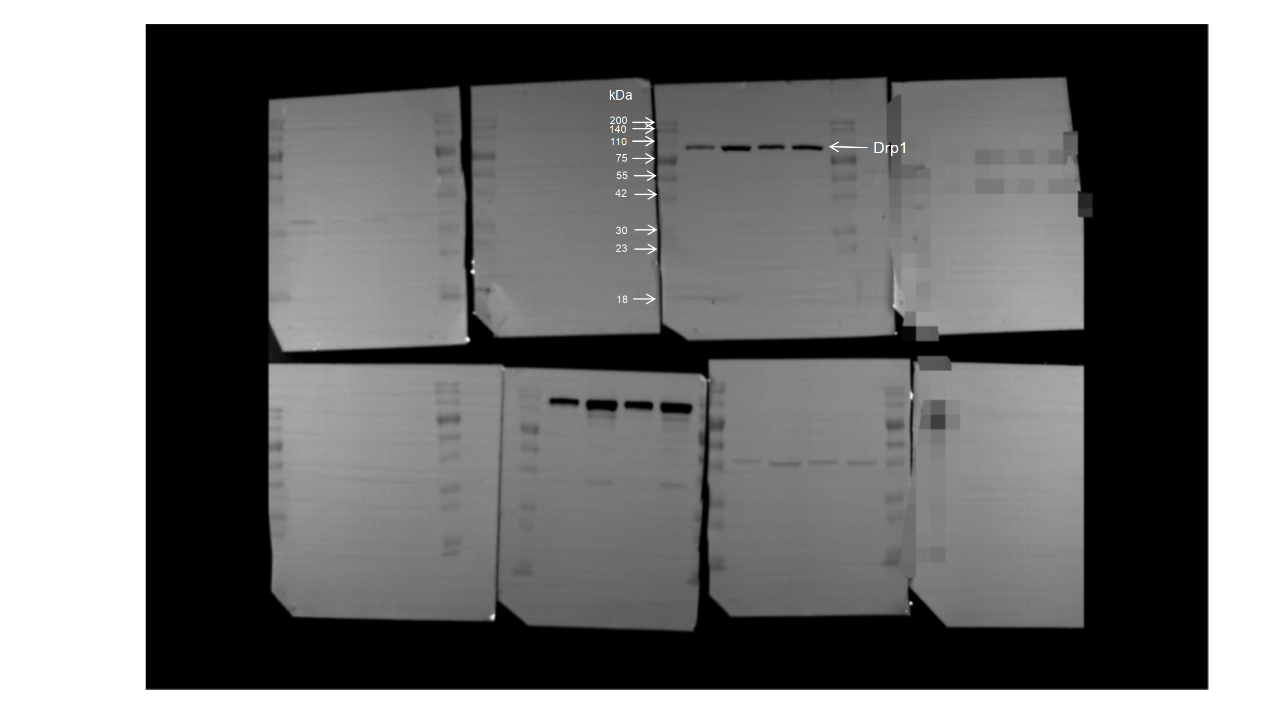


Mfn2 (80 kDa)


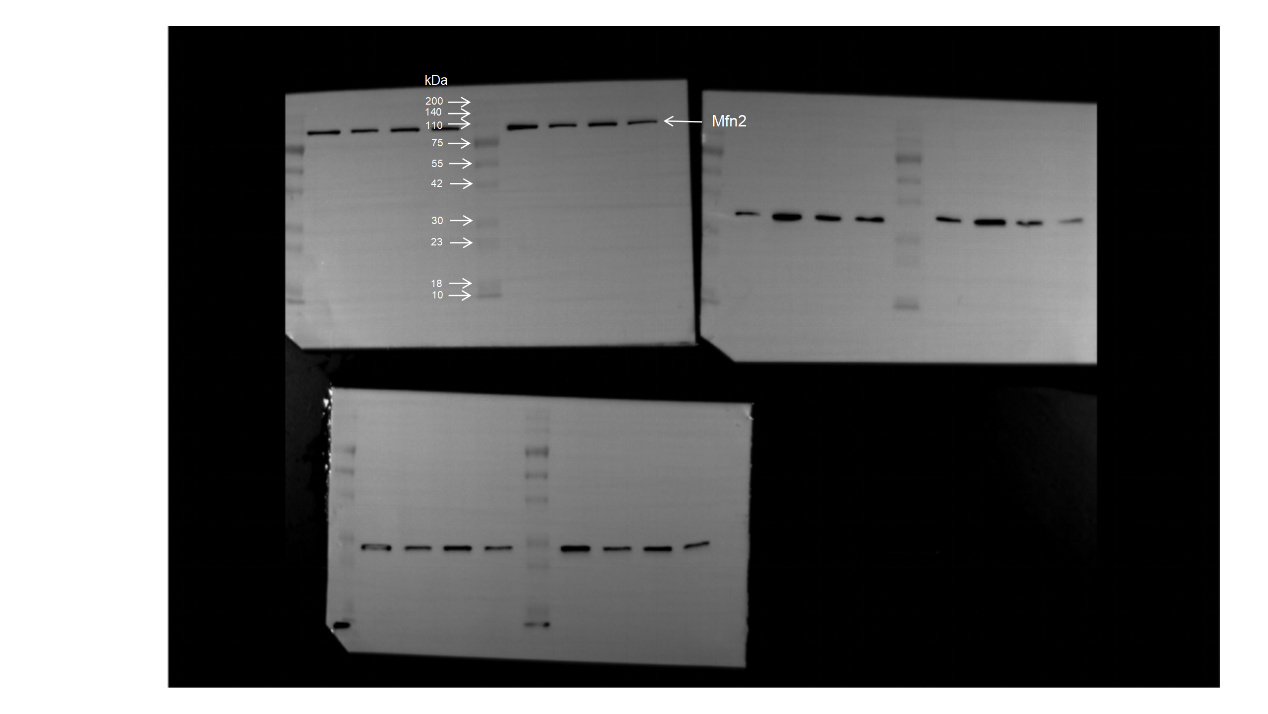


β-Actin (45 kDa)


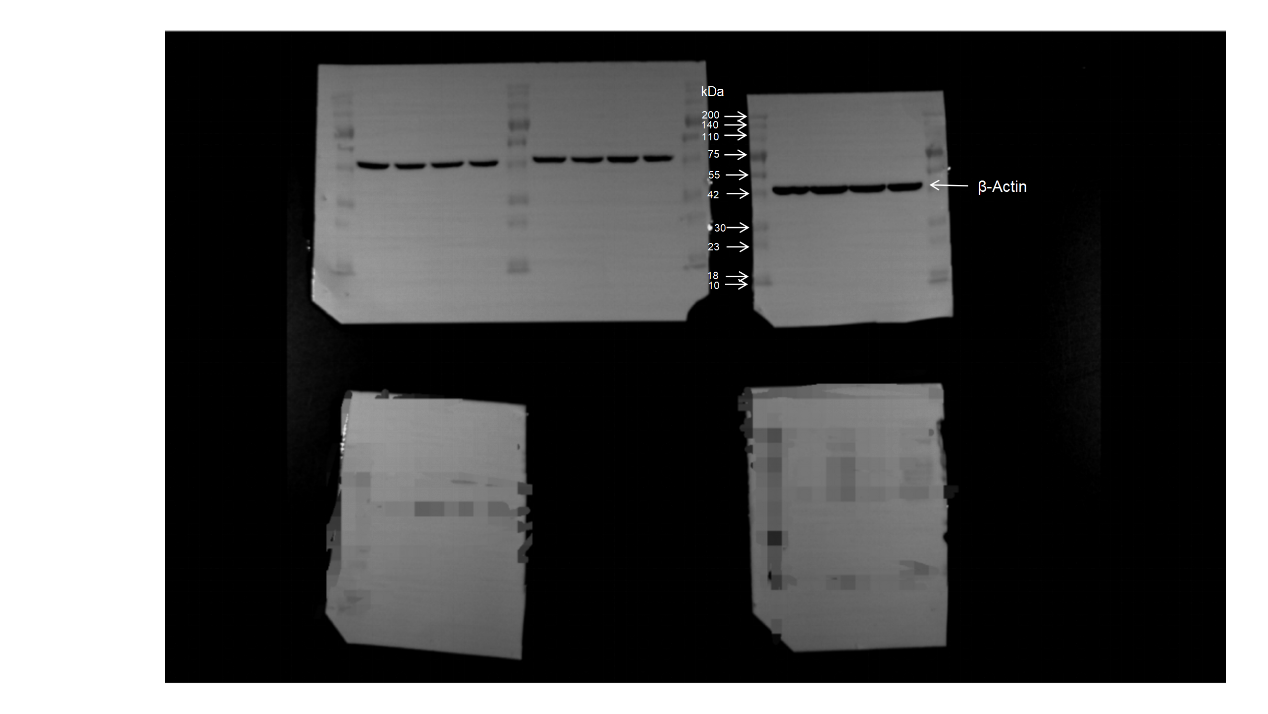


Fig.3

Tnmd (37 kDa)


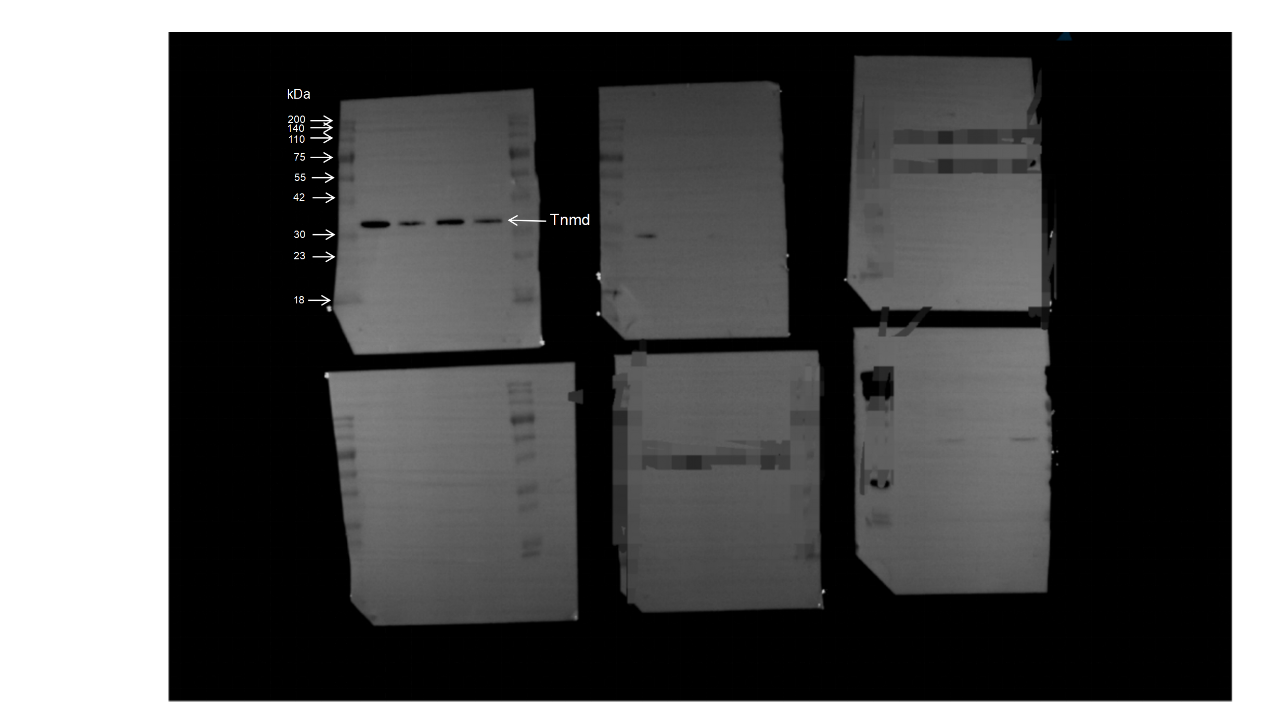


β-Actin (45 kDa)


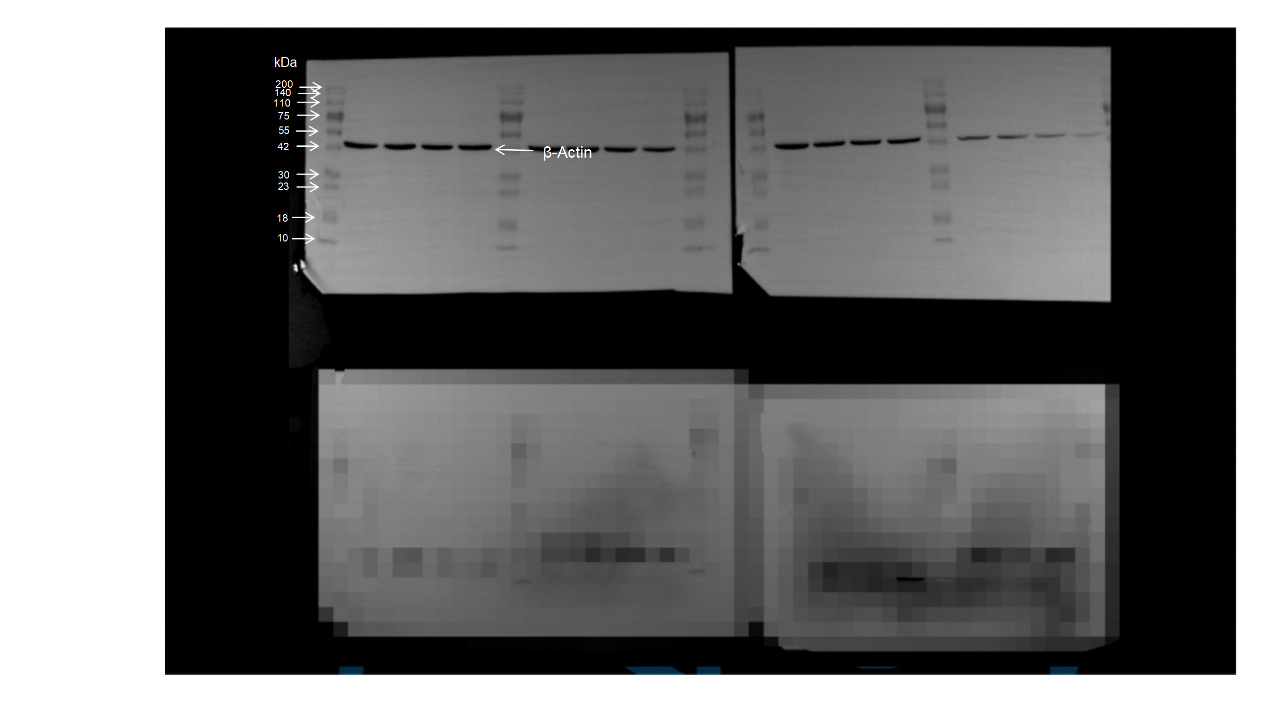


MMP-1 (54 kDa)


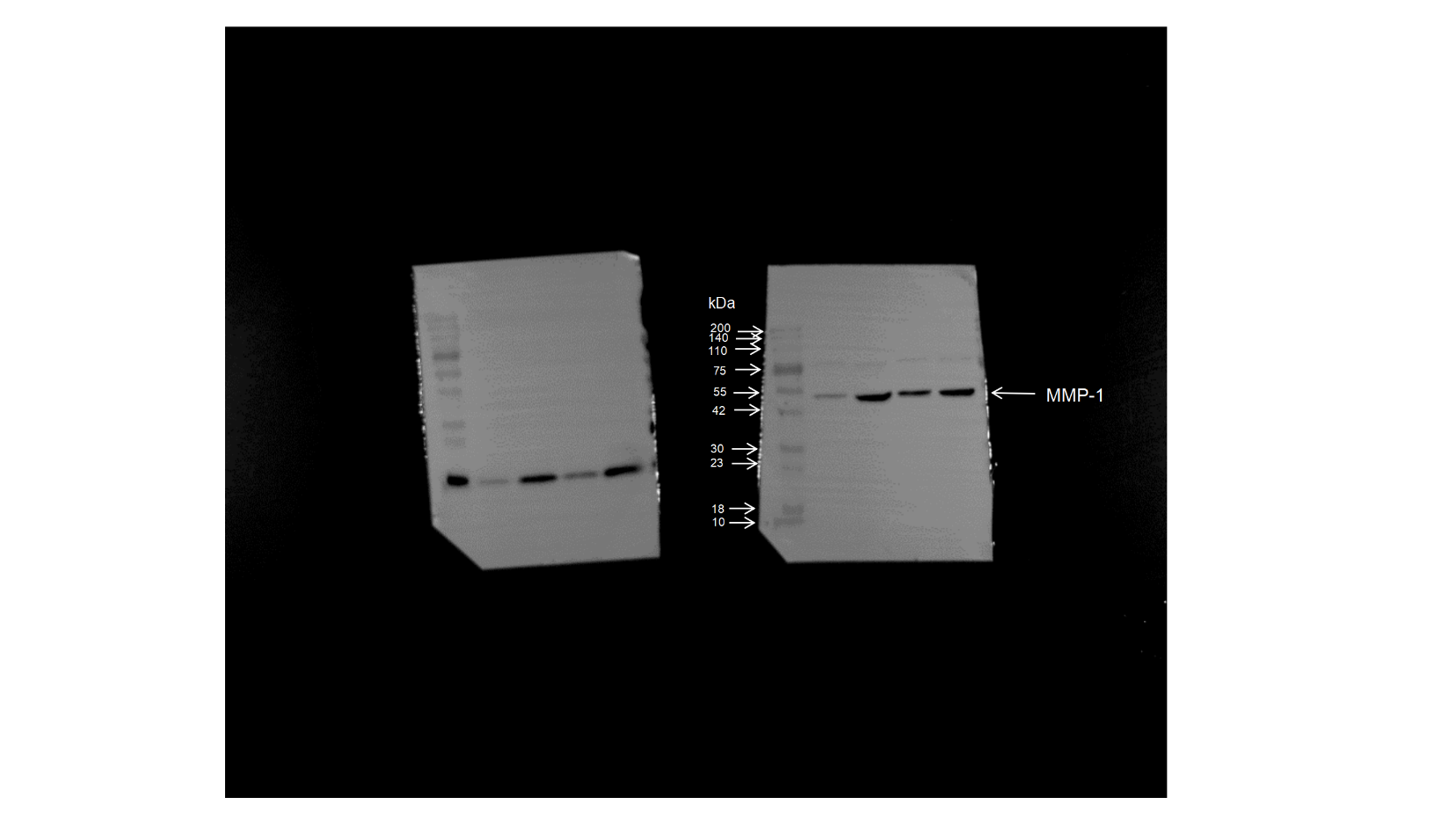


β-Actin (45 kDa)


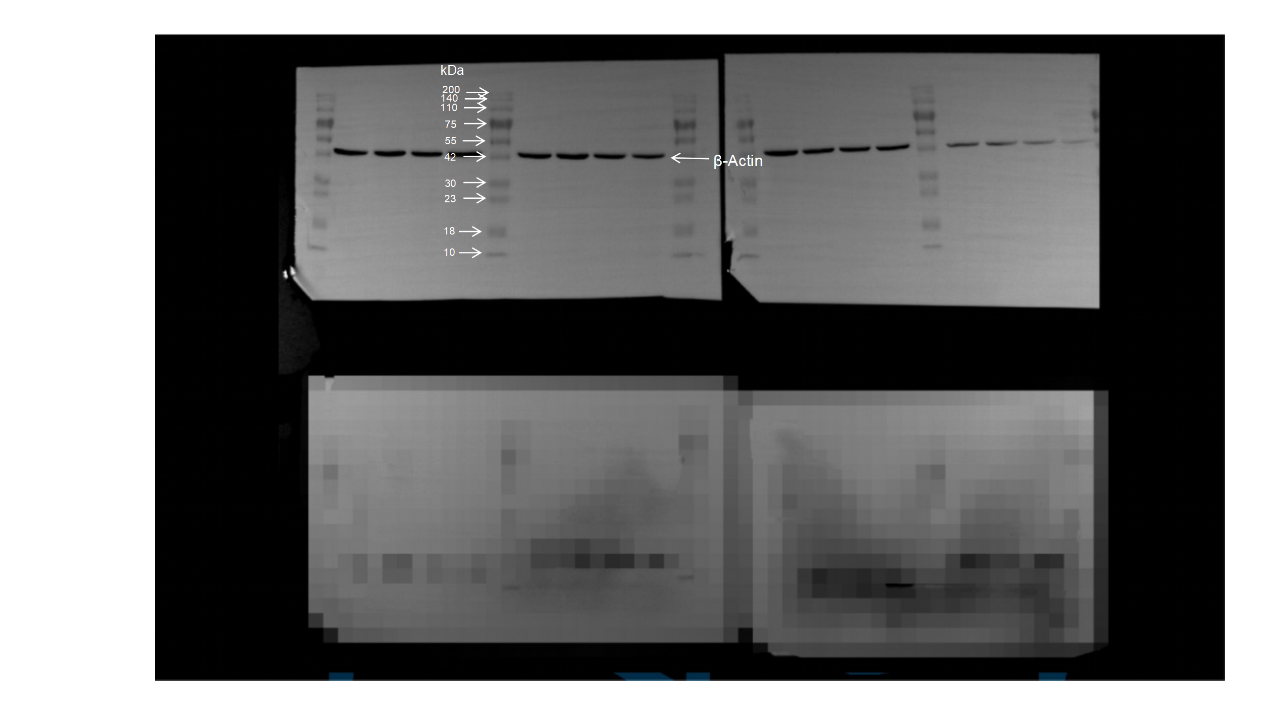


Col I (120 kDa)


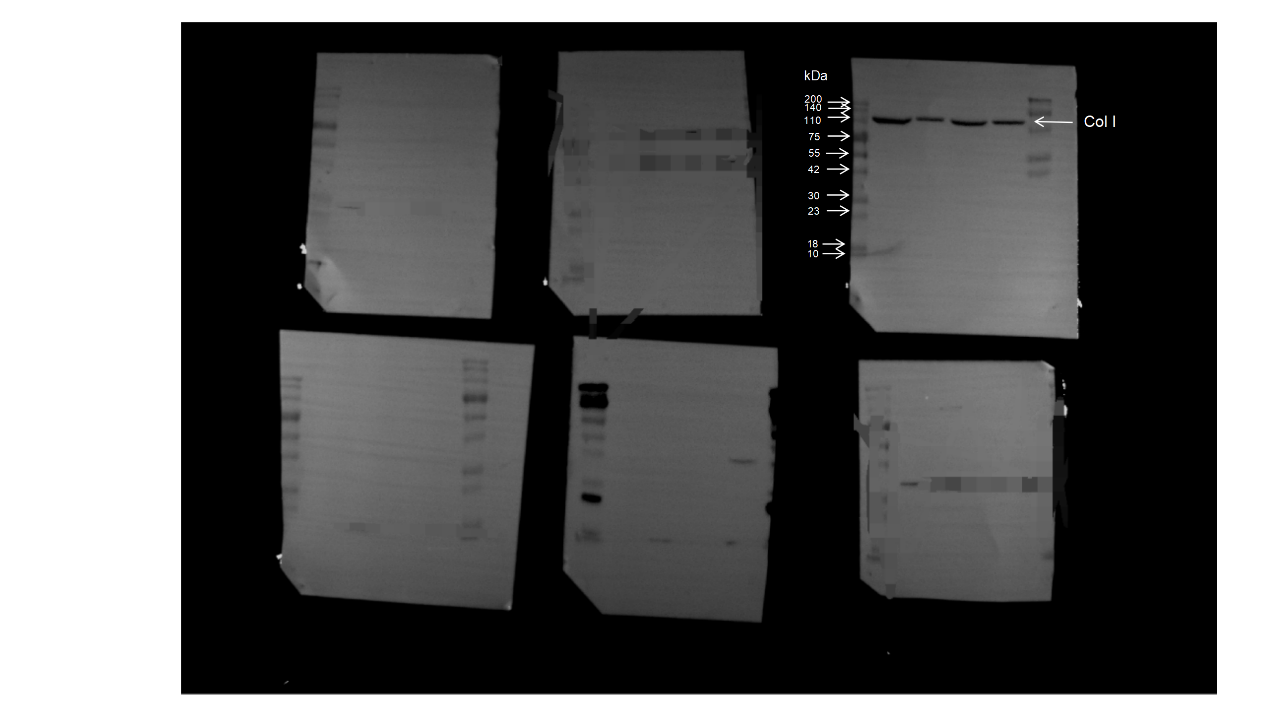


Col III (120 kDa)


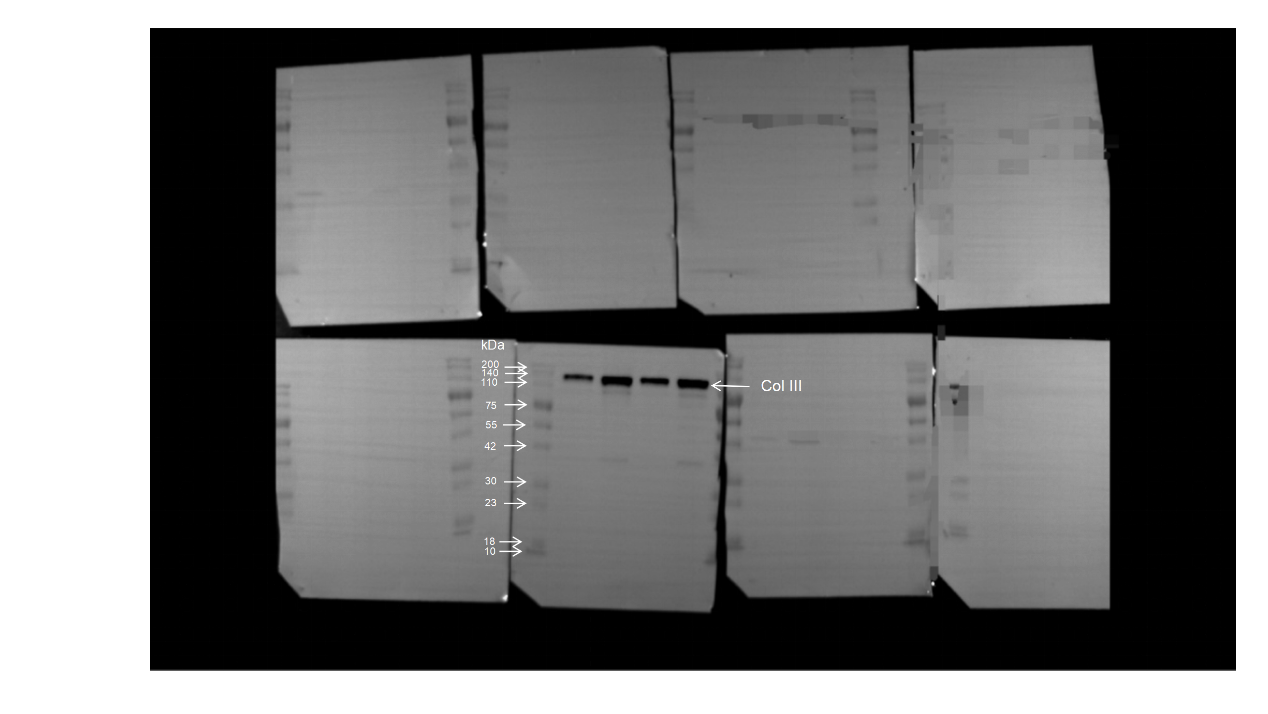


β-Actin (45 kDa)


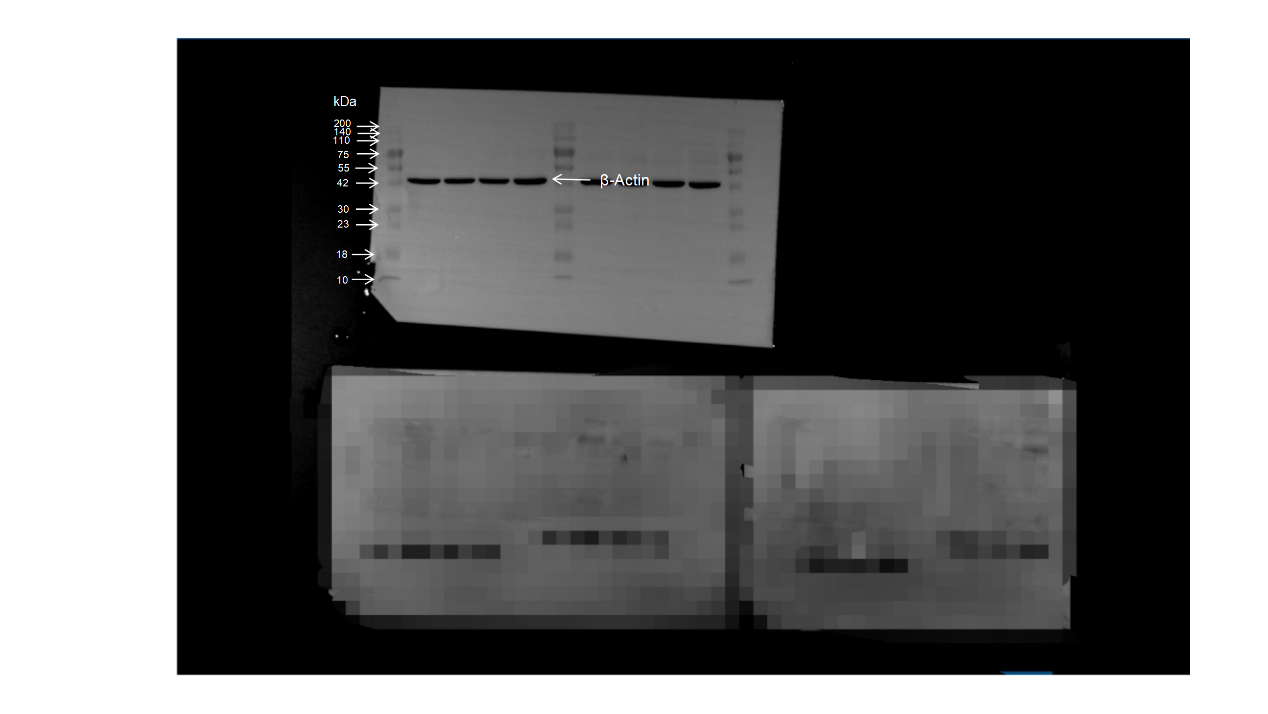

Supplement: Supplementary file 7 — Additional file 7. The original gel/blot images are displayed. [file 13287_2023_3329_MOESM7_ESM.docx]
